# In vivo monitoring of remnant undifferentiated neural cells following human induced pluripotent stem cell‐derived neural stem/progenitor cells transplantation

**DOI:** 10.1002/sctm.19-0150

**Published:** 2020-01-06

**Authors:** Yuji Tanimoto, Tomoteru Yamasaki, Narihito Nagoshi, Yuichiro Nishiyama, Satoshi Nori, Soraya Nishimura, Tsuyoshi Iida, Masahiro Ozaki, Osahiko Tsuji, Bin Ji, Ichio Aoki, Masahiro Jinzaki, Morio Matsumoto, Yasuhisa Fujibayashi, Ming‐Rong Zhang, Masaya Nakamura, Hideyuki Okano

**Affiliations:** ^1^ Department of Physiology Keio University School of Medicine Tokyo Japan; ^2^ Department of Orthopaedic Surgery Keio University School of Medicine Tokyo Japan; ^3^ Department of Advanced Nuclear Medicine Sciences National Institute of Radiological Sciences, Quantum Medical Science Directorate, National Institutes for Quantum and Radiological Science and Technology (QST) Chiba Japan; ^4^ Department of Functional Brain Imaging National Institute of Radiological Sciences, Quantum Medical Science Directorate, National Institutes for Quantum and Radiological Science and Technology (QST) Chiba Japan; ^5^ Institute for Quantum Life Science, National Institutes for Quantum and Radiological Science and Technology (QST) Chiba Japan; ^6^ Department of Radiology Keio University School of Medicine Tokyo Japan

**Keywords:** human‐induced pluripotent stem cell‐derived neural stem/progenitor cells, in vivo imaging, PET, stem cell transplantation, spinal cord injury

## Abstract

Transplantation of human‐induced pluripotent stem cell‐derived neural stem/progenitor cells (hiPSC‐NS/PCs) is a promising treatment for a variety of neuropathological conditions. Although previous reports have indicated the effectiveness of hiPSC‐NS/PCs transplantation into the injured spinal cord of rodents and nonhuman primates, long‐term observation of hiPSC‐NS/PCs post‐transplantation suggested some “unsafe” differentiation‐resistant properties, resulting in disordered overgrowth. These findings suggest that, even if “safe” NS/PCs are transplanted into the human central nervous system (CNS), the dynamics of cellular differentiation of stem cells should be noninvasively tracked to ensure safety. Positron emission tomography (PET) provides molecular‐functional information and helps to detect specific disease conditions. The current study was conducted to visualize Nestin (an NS/PC marker)‐positive undifferentiated neural cells in the CNS of immune‐deficient (nonobese diabetic‐severe combined immune‐deficient) mice after hiPSC‐NS/PCs transplantation with PET, using 18 kDa translocator protein (TSPO) ligands as labels. TSPO was recently found to be expressed in rodent NS/PCs, and its expression decreased with the progression of neuronal differentiation. We hypothesized that TSPO would also be present in hiPSC‐NS/PCs and expressed strongly in residual immature neural cells after transplantation. The results showed high levels of TSPO expression in immature hiPSC‐NS/PCs‐derived cells, and decreased TSPO expression as neural differentiation progressed in vitro. Furthermore, PET with [^18^F] FEDAC (a TSPO radioligand) was able to visualize the remnant undifferentiated hiPSC‐NS/PCs‐derived cells consisting of TSPO and Nestin^+^ cells in vivo. These findings suggest that PET with [^18^F] FEDAC could play a key role in the safe clinical application of CNS repair in regenerative medicine.

## Graphical abstract

By utilizing the characteristic of neural stem/progenitor cells (NS/PCs) to express translocator protein (TSPO), positron emission tomography (PET) with TSPO ligand was able to visualize residual immature neural cells after NS/PCs transplantation.

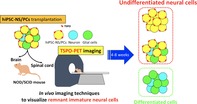

**Significance statement**

Stem cell‐based therapy using neural stem/progenitor cells (NS/PCs) derived from human‐induced pluripotent cells (hiPSCs) provides a promising approach for treating neurodegenerative diseases and neurotrauma. However, not all transplanted cells fully differentiate into mature neurons and glial cells, even if clinically “safe” clones are used. These undifferentiated cells can trigger tumorigenic overgrowth due to their pluripotency. By utilizing the characteristic of NS/PCs to express the 18 kDa translocator protein (TSPO), positron emission tomography with TSPO ligand was able to visualize residual immature neural cells after NS/PCs transplantation into central nervous system and could potentially have critical importance in regenerative medicine.

## 1 INTRODUCTION

Stem cell‐based technology, such as transplantation of neural stem cell/progenitor cells (NS/PCs) derived from embryonic stem cells (ESCs) or induced pluripotent cells (iPSCs), provides a promising approach in neuroregenerative medicine.^1^, ^2^, ^3^, ^4^ Transplanted “safe” NS/PCs have been found to fully differentiate into three lineages (neuron, astrocyte, and oligodendrocyte), contributing to the recovery of locomotor function in traumatic brain injury (TBI) and spinal cord injury (SCI) in experimental animal models.^5^, ^6^, ^7^ Long‐term observation of transplanted cells, however, has revealed the existence of “unsafe” NS/PCs characterized by differentiation‐resistant properties that could cause abnormal cell growth in the injured spinal cords of rodents due to residual immature neuronal cells after hiPSC‐NS/PCs transplantation.^8^, ^9^ Therefore, it is necessary to select “safe” and “unsafe” clones in the cell manufacturing process.^7^, ^10^, ^11^ Furthermore, since the extent of cellular differentiation and maturation depends on the host microenvironment,^12^ even “safe” clones, which can be purified for homogeneity and produced as high‐quality populations, may not be able to completely differentiate into neural cell types at the transplanted site. Therefore, clinical application of hiPSC‐NS/PCs to central nervous system (CNS) disorders would benefit from a relatively noninvasive detection technique for monitoring the progress of cellular‐differentiation even after “safe” clone transplantation.

Among various types of in vivo imaging techniques, positron emission tomography (PET) is a useful modality because it provides molecular‐functional information with high sensitivity.^13^, ^14^, ^15^ In addition, longitudinal PET imaging could enable monitoring of dynamic changes of a target molecule in vivo.

The 18 kDa translocator protein (TSPO), also known as a peripheral‐type benzodiazepine receptor,^16^ is mainly expressed in an outer mitochondrial membrane, and its expression is upregulated by specific types of neoplastic cells such as gliomas,^13^, ^17^ activated microglia,^18^, ^19^, ^20^ and reactive astrocytes^21^ associated with neuroinflammation. Additionally, it has been suggested that TSPO could be a hallmark of cellular‐differentiation in NS/PCs analogous to Nestin, which is widely known as an NS/PC marker.^22^, ^23^ Therefore, we focused on visualizing TSPO expression in remnant undifferentiated hiPSC‐NS/PC‐derived neural cells using the PET modality.

In the present study, in order to determine the efficiency of PET with TSPO ligand, we used two different types of iPSC‐derived NS/PCs; “unsafe” 253G1‐NS/PCs known to have differentiation‐resistant proliferative properties^8^, ^9^ and “safe” 414C2‐NS/PCs, which were reported as a nontumorigenic hiPSC‐NS/PCs.^11^ First, we examined differences in TSPO expression levels in each hiPSC‐NS/PCs before and after neuronal induction using immunohistochemistry, real‐time reverse‐transcription polymerase chain reaction (RT‐PCR) and Western blot assays in vitro. Next, we transplanted each hiPSC‐NS/PCs into the brain and spinal cord of nonobese diabetic‐severe combined immune‐deficient (NOD‐SCID) mice and performed PET imaging with *N*‐benzyl‐*N*‐methyl‐2‐[7,8‐dihydro‐7‐(2‐[^18^F]fluoroethyl)‐8‐oxo‐2‐phenyl‐9H‐purin‐9‐yl] acetamide ([^18^F] FEDAC), a clinically applicable TSPO‐selective radioligand.^19^ In an in vitro study, TSPO was initially expressed in both hiPSC‐NS/PCs types, but decreased over time as neural differentiation progressed in “safe” 414C2‐NS/PCs. Notably, “unsafe” 253G1‐NS/PCs exhibited a differentiation‐resistant profile and continued to express high levels of TSPO expression in vitro. Consistent with these results, PET with [^18^F] FEDAC was able to detect the poorly differentiated neural tissues of 253G1‐NS/PCs‐grafted mice brains in vivo due to their high TSPO density. These results suggest that PET imaging for TSPO provides an appropriate method for monitoring the dynamics of neural differentiation and maturation following hiPSC‐NS/PCs transplantation by observing remnant immature cells in clinical settings. This approach has the potential to ensure the safety of NS/PCs‐based treatment in humans in the future.

## 2 MATERIALS AND METHODS

### 2.1 Cell culture, hiPSC‐NS/PCs‐derived NS/PCs formation assay, neural induction, and lentivirus transduction

The methods used to culture the human male glioblastoma multiforme U‐251MG cells and the hiPSCs (253G1‐NS/PCs and 414C2‐NS/PCs) and to induce neural differentiation were performed as previously described.^2^, ^5^, ^24^, ^25^, ^26^ In the analyses of neuronal differentiation, hiPSC‐NS/PCs were plated onto poly‐l‐ornithine/fibronectin‐coated 48‐well chamber slides (Costar 3548; Corning, New York) at a density of 1 × 10^5^ cells/mL and cultured in medium without growth factors at 37°C in 5% CO_2_ and 95% air for 14 days. Differentiated cells were fixed paraformaldehyde (PFA) in 0.1 phosphate‐buffered saline (PBS) and stained with the following primary antibodies for immunocytochemistry: anti‐human‐specific TSPO (NP157, rabbit IgG, 1:300; National Institute for Quantum and Radiological Science and Technology, Chiba, Japan), antihuman‐specific Nestin protein (MAB5236, mouse IgG1, 1:500; Merck Millipore, Billerica, Massachusetts), anti‐β III‐tubulin (T8660, mouse IgG2b, 1:500; Sigma‐Aldrich, St. Louis, Missouri), anti‐NeuN (MAB377, mouse IgG1, 1:500; Merck Millipore). Nuclei were stained with Hoechst 33258 (10 μg/mL, Sigma‐Aldrich). All in vitro images were obtained using confocal laser scanning microscopy (LCM 700; Carl Zeiss, Jenna, Germany).

### 2.2 Real‐time reverse‐transcription polymerase chain reaction

RNA isolation and RT‐PCR were performed as described previously.^5^ Detailed methods are presented in Supporting Information (SI) Materials and Methods.

### 2.3 Western blotting assay

Protein isolation and Western blotting assay were performed as described previously.^27^ Detailed methods are presented in SI Materials and Methods.

### 2.4 Transplantation

Transplantation was performed as described previously.^8^, ^28^ Detailed methods are presented in SI Materials and Methods. All experiments were performed in accordance with the Guidelines for the Care and Use of Laboratory Animals of Keio University (Assurance No. 13020) and the Guide for the Care and Use of Laboratory Animals.

### 2.5 Bioluminescence imaging

Bioluminescence imaging (BLI) was performed as described previously, with slight modifications.^24^ Detailed methods are presented in SI Materials and Methods.

### 2.6 PET and computed tomography scanning

PET and computed tomography (CT) was performed as described previously.^19^ Detailed methods are presented in SI Materials and Methods.

### 2.7 Production of [^18^F] FEDAC

Radiosynthesis of [^18^F] FEDAC was conducted in accord with a previous report.^19^ Detailed methods are presented in SI Materials and Methods.

### 2.8 Magnetic resonance imaging

Magnetic resonance imaging (MRI) was performed as described previously.^29^ Detailed methods are presented in SI Materials and Methods.

### 2.9 Ex vivo autoradiography

Ex vivo autoradiography was performed as described previously.^30^ Detailed methods are presented in SI Materials and Methods.

### 2.10 Histological analyses

After autoradiography, the brains and spinal cords were used for histological analyses. Detailed methods are presented in SI Materials and Methods.

### 2.11 Statistical analysis

All data are presented as means ± SD. One‐way analysis of variance (ANOVA) followed by the Tukey‐Kramer test for multiple comparisons was used for neuronal differentiation analyses, RT‐PCR gene expression profile analyses, Western blot analyses, and ipsilateral‐to‐contralateral ratio (ICR) for autoradiography. Student's paired *t* tests were used for calculating the AUC for the PET data. One‐way ANOVA followed by Dunnett's test for multi comparisons was used for immunohistochemistry results for the portion of TSPO^+^ cells. *P*‐values <.05, <.01, or .001 were considered to indicate statistical significance. Microsoft Excel 2016 and IBM SPSS Statistics (ver. 24) were used for all calculations.

## 3 RESULTS

### 3.1 TSPO was expressed in hiPSC‐NS/PCs, and downregulated over time as neuronal differentiation progressed in vitro

**Figure 1:**
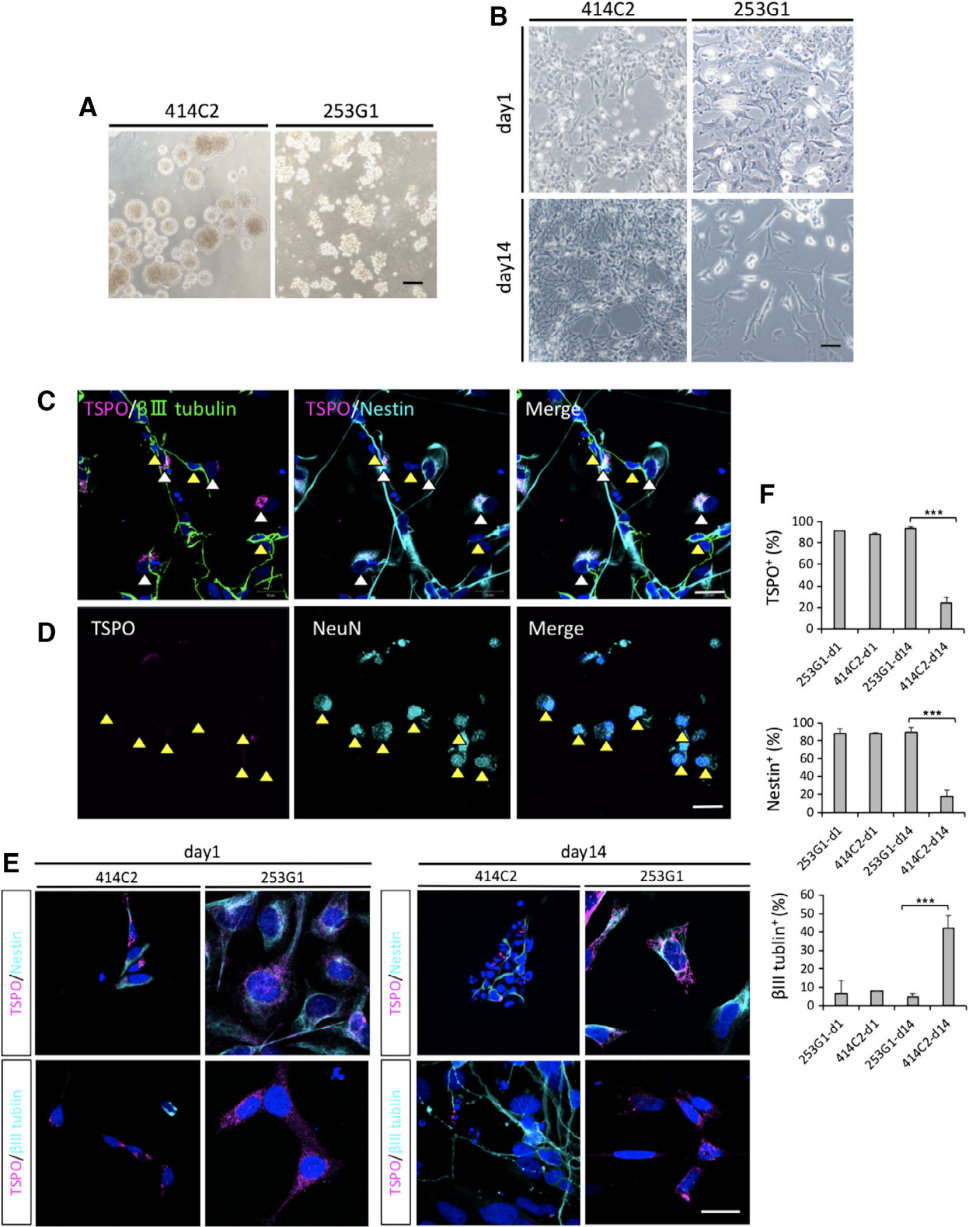
Immunohistochemistry results of the in vitro differentiation of hiPSC‐NS/PCs revealed different levels of TSPO expression between 253G1‐ and 414C2‐NS/PCs during neuronal induction. A, Representative images of micrographs showing hiPSC‐NS/PCs aggregates for each group and cell line. B, Representative images of micrographs before and after hiPSC‐NS/PCs neural differentiation. C‐F, hiPSC‐NS/PCs dissociated into single cells, seeded on coverglasses, and immunostained with TSPO, β III‐tubulin (a neuronal marker), Nestin (an NS/PC marker), NeuN (a mature neuronal marker). C, D, Fourteen days after neuronal induction of 414C2‐NS/PCs (414C2‐d14), sections were triple‐stained with TSPO (red), β III tubulin (green), and Nestin (cyan blue). Most cells coexpressed TSPO and Nestin (white arrow heads) contrary to β III tubulin^+^ cells which did not express significant amounts of TSPO (yellow arrow heads) (C). 414C2‐d14 sections were stained with TSPO (red), NeuN (cyan blue) (yellow arrowheads indicate TSPO^−^ and NeuN^+^ cells) (D). The nuclei were stained with Hoechst 33258. E, Representative staining results for each hiPSC‐NS/PCs during neuronal differentiation, comparing d1 and d14. The nuclei were stained with Hoechst 33258. F, Bar graph showing the percentage of TSPO^+^, Nestin^+^, and β III tubulin^+^ cells. Values are mean ± SD (n = 3, for each cell type). ***P < .001. Scale bars = 200 μm in (A), 50 μm in (B), 20 μm in (C‐E). hiPSC‐NS/PCs, human‐induced pluripotent stem cell‐derived neural stem/progenitor cells

We first examined the levels of TSPO expression in two human iPSC lines (414C2 and 253G1) derived from NS/PCs, and their differentiation‐induced cells in vitro. As neuronal differentiation progressed, 414C2‐NS/PCs were observed to exhibit a more complete neural network compared to 253G1‐NS/PCs (Figure 1A,B). Subsequent immunohistochemical analyses performed using TSPO, β‐tubulin isotype III (β III tubulin; a neuronal marker) and human Nestin (an NS/PC marker) showed TSPO expression in both types of hiPSC‐NS/PCs. Most TSPO^+^ cells were also Nestin^+^, whereas TSPO expression was reduced in β III tubulin^+^ cells as neuronal differentiation progressed (Figure 1C). In addition, NeuN (a mature neuronal marker) ^+^ cells did not show any detectable TSPO expression (Figure 1D).

Next, we performed quantitative analyses to determine the different levels of TSPO expression among each hiPSC‐NS/PCs‐derived cell line cultured for 1 day (d1) or 14 days (d14) (Figure 1E,F). In the 414C2‐d14 group, the percentages of TSPO^+^ and Nestin^+^ cells were 23.8 ± 5.6% and 18.2 ± 7.1%, which were significantly lower than those of the 253G1‐d14 (TSPO^+^ cells: 93.8 ± 1.4%, *P* < .001; Nestin^+^ cells: 90.1 ± 5.0%, *P* < .001) and 414C2‐d1 (TSPO^+^ cells: 88.6 ± 0.8%, *P* < .001; Nestin^+^ cells: 88.6 ± 0.6%, *P* < .001) cells. However, the percentage of β III tubulin^+^ neurons in 414C2‐d14 cells (42.2 ± 6.7%) was significantly higher than 253G1‐d14 cells (4.6 ± 2.1%, *P* < .001).

### 3.2 Undifferentiated/differentiation‐resistant hiPSC‐NS/PCs‐derived neuronal cells highly expressed TSPO mRNA and protein

**Figure 2:**
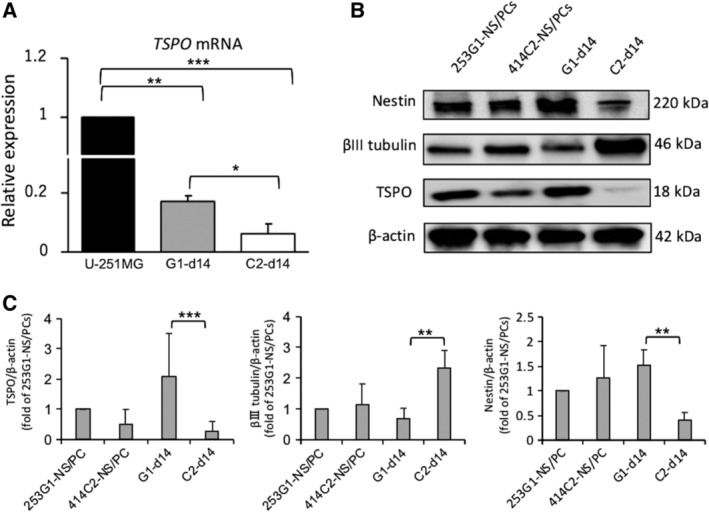
RT‐PCR and Western blot results of the 253G1‐NS/PCs demonstrating a differentiation‐resistant profile and high levels of TSPO even after inducing differentiation in vitro. A, RT‐PCR results of the expression of *TSPO* mRNA of 253G1‐ and 414C2‐NS/PCs before and after neuronal differentiation, relative to the U‐251MG (control) group (black bar). The data were normalized to the reference *GAPDH* levels. Values are means ± SD (n = 3, each). B, Western blot results of the expression of TSPO, β III tubulin, and Nestin protein levels of 253G1‐ and 414C2‐NS/PCs before and after neuronal differentiation using Western blot. C, Quantitative analysis of TSPO, Nestin, and β III tubulin and protein levels using Western blot. The data were normalized to the reference β*‐*actin levels. The relative intensities on the band of 414C2‐NS/PCs, 253G1‐d14, and 414C2‐d14 were compared to the 253G1‐NS/PCs. Values are mean ± SD (n = 4, each). *P < .05, **P < .01, ***P < .001. NS/PCs, neural stem/progenitor cells

RT‐PCR was performed to assess the levels of TSPO mRNA for each hiPSC‐NS/PCs type after neuronal induction. The data were presented as expression levels relative to the U‐251MG (human brain glioblastoma cell line; GBM). Since previous studies reported that GBM strongly expressed TSPO,^13^ we used U‐251MG as a positive control in RT‐PCR and PET experiments (Figure 2A). The expression of TSPO mRNA in the U‐251MG group was significantly higher than that in the 253G1‐d14 (*P* < .01) and 414C2‐d14 groups (*P* < .001). Importantly, the results revealed significantly elevated levels of TSPO mRNA in the 253G1‐d14 group compared to the 414C2‐d14 group (*P* < .05).

Next, Western blot analysis was performed to examine the protein levels of TSPO in each hiPSC‐NS/PCs before and after neuronal differentiation (Figure 2B,C). Consistent with the results of RT‐PCR, TSPO protein levels in the 253G1‐d14 group were significantly higher than those in the 414C2‐d14 group (*P* < .001), while maintaining high levels of Nestin expression (*P* < .01). In contrast, TSPO and Nestin protein levels in the 414C2‐d14 group strongly decreased over time with an increase in β III tubulin expression.

### 3.3 PET with [^18^F] FEDAC showed strongly radioactive accumulation corresponding to the engraftment of 253G1‐NS/PCs after transplantation

To visualize remnant immature neural cells after hiPSC‐NS/PCs transplantation using PET with [^18^F] FEDAC, we transplanted 5 × 10^5^ cells, including 253G1‐NS/PCs, 414C2‐NS/PCs, U‐251MG (positive control), or PBS (negative control) into the right striatum of NOD‐SCID mice. We chronologically monitored the engraftment of transplanted cells using BLI. These cells were lentivirally transduced with ffLuc, a fusion protein between a fluorescent Venus protein and a firefly luciferase,^31^ which allowed us to monitor the growth of the grafted cells by their fluorescent Venus signals and bioluminescent luciferase signals. The photon counts of the 253G1 group increased more rapidly than that of the 414C2 group over time (Figure S1).

**Figure 3:**
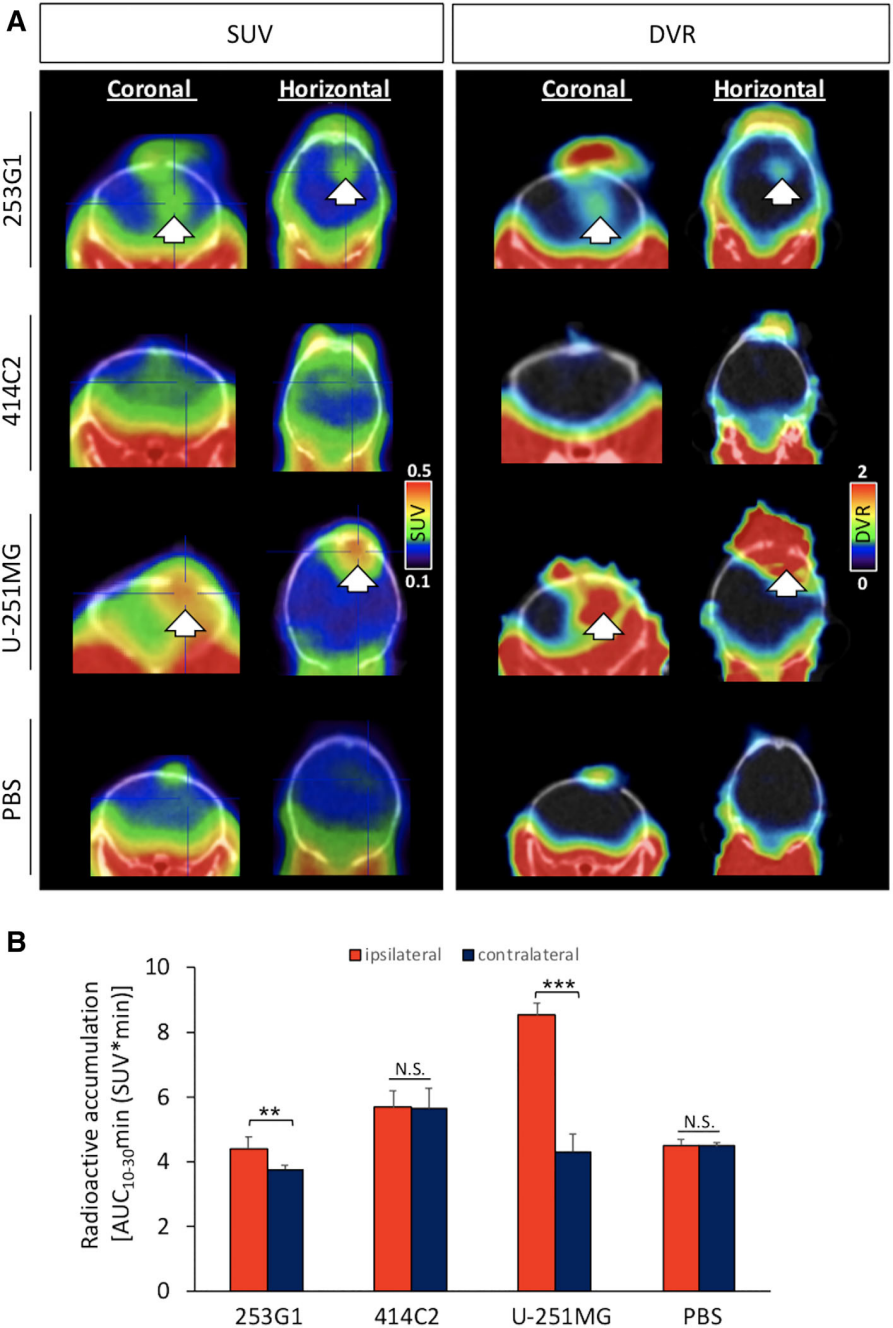
[^18^F] FEDAC‐PET detecting the 253G1‐NC/PCs grafted area in vivo. A‐D, Representative [^18^F] FEDAC‐PET/CT fusion images (coronal and horizontal images) in hiPSC‐NS/PCs‐, U‐251MG‐, and PBS‐grafted mouse brains. Left side: SUV images acquired between 10 and 30 minutes. Right side: dynamic reconstructed DVR images acquired between 10 and 30 minutes. Arrows indicate the transplanted site. B, Area under the curves (AUC_10‐30 min_; SUV × min) of [^18^F] FEDAC were calculated from the time‐activity curves between 10 and 30 minutes after injection of [^18^F] FEDAC (red bar: ipsilateral side, ie, the injected site; blue bar: contralateral side, ie, the intact site). Values are means ± SD (n = 5, 4, 5, and 4, respectively). **P < .01, ***P < .001, N.S., not significant. DVR, distribution volume ratio; hiPSC‐NS/PCs, human‐induced pluripotent stem cell‐derived neural stem/progenitor cells; PBS, phosphate‐buffered saline; SUV, standard uptake volume

Dynamic small‐animal PET scanning using [^18^F] FEDAC was performed at four to eight weeks after transplantation. The timing of PET evaluation was dependent on the health of the mice during the experimental period. Only the U‐251MG group was scanned with PET two weeks after transplantation because the life span of these animals was between 2 and 4 weeks. Importantly, representative PET images, acquired by summing up the [^18^F] FEDAC signal generated between 10 and 30 minutes and scaled with distribution volume ratio (DVR), a quantitative index for specific biding, could detect radioactive accumulation in the transplanted site (the right striatum) of the 253G1 and U‐251MG groups as opposed to the contralateral side (Figure 3A; Table S1). The 414C2 and PBS groups showed no detectable signal in both sides. We calculated the uptake of radioactivity between 10 and 30 minutes after the injection, represented as the area under the curve (AUC_10‐30 min_) in the ipsilateral and contralateral sides of each mouse brain (Figure 3B). In the 253G1 and U‐251MG groups (n = 5, each), the AUC_10‐30 min_ of the ipsilateral side (the transplanted side; 4.4 ± 0.4 and 8.5 ± 0.4, respectively) was significantly higher than that of contralateral side (the intact side; 3.8 ± 0.1 and 4.3 ± 0.6, respectively) (253G1 group, *P* < .01 and U‐251MG group, *P* < .001).

**Figure 4:**
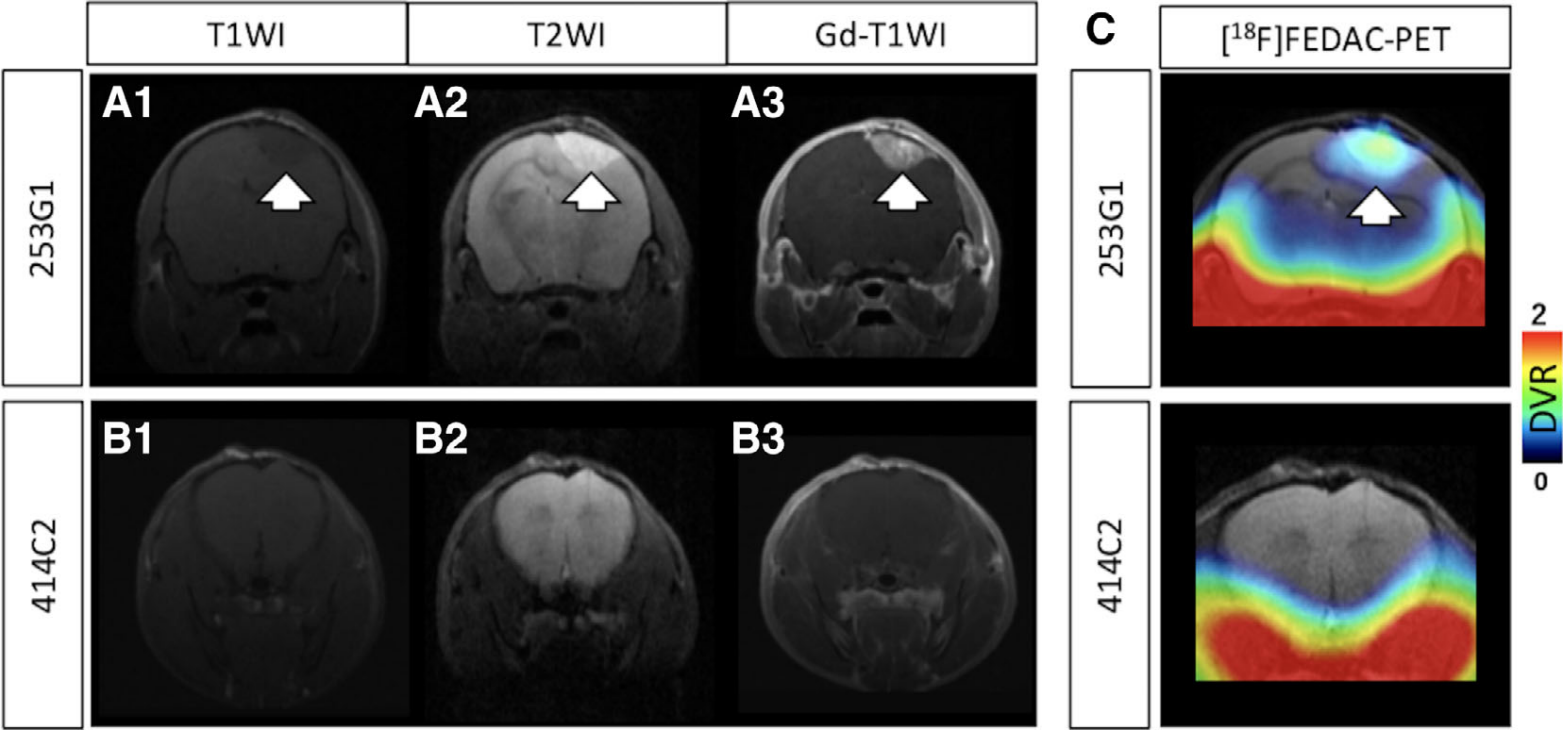
Comparison of [^18^F] FEDAC‐PET and gadolinium‐enhanced MRI. A,B, Representative MRI images of in each hiPSC‐NS/PCs grafted mouse coronal brain. T1‐weighted imaging (A‐1, B‐1); T2‐weighted imaging (A‐2, B‐2); and gadolinium enhanced T1‐weighted imaging (A‐3, B‐3). C, Corresponding [^18^F] FEDAC‐PET/MRI fusion images of each hiPSC‐NS/PCs transplanted mouse coronal brain. Arrows indicate the transplanted 253G1‐NS/PCs depicted by MRI and were transferred to PET images. hiPSC‐NS/PCs, human‐induced pluripotent stem cell‐derived neural stem/progenitor cells; MRI, magnetic resonance imaging; PET, positron emission tomography

Next, to confirm whether the radioactive signals in [^18^F] FEDAC‐PET images in each hiPSC‐NS/PCs‐transplanted mouse corresponded with the lesion area after transplantation, we performed contrast‐enhanced MRI using gadolinium‐based contrast agent. In the 253G1‐NS/PCs‐grafted mouse brain, the contrast‐enhanced areas were detected on the gadolinium injected T1‐weighted MR images (Figure 4A). Corresponding PET images revealed intense DVR signals of [^18^F] FEDAC uptake in the same lesion (Figure 4C). In contrast, 414C2‐NS/PCs‐grafted mice brains did not exhibit any detectable signal enhancement in both T1 and T2‐weighted images on MRI and PET (Figure 4B,C).

### 3.4 The radioactive accumulation in PET images was supported using ex vivo autoradiography

**Figure 5:**
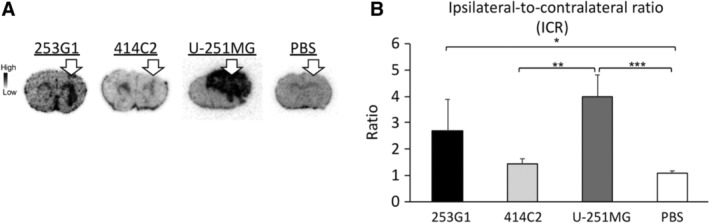
Ex vivo autoradiography showing the selective uptake of [^18^F] FEDAC in the 253G1‐NS/PCs grafted mouse brains. A, Representative ex vivo autoradiographs of the [^18^F] FEDAC on the 253G1‐NS/PCs‐, 414C2‐NS/PCs‐, U‐251MG‐, or PBS‐grafted mouse brain sections. Arrows indicate the transplant site. B, The uptake ratios of radioactivity between ipsilateral and contralateral sides of each mouse brain were calculated as ICRs. Bar graph showing the ICR of the 253G1, 414C2, U‐251MG, and PBS groups. Values are mean ± SD (n = 5, 4, 5, and 4, respectively). *P < .05, **P < .01, ***P < .001. ICR, ipsilateral‐to‐contralateral ratio; NS/PCs, neural stem/progenitor cells; PBS, phosphate‐buffered saline

To examine the detailed anatomical investigation of [^18^F] FEDAC‐binding sites in the hiPSC‐NS/PCs‐grafted brains, we performed ex vivo autoradiography with [^18^F] FEDAC (Figure 5A). The 253G1, 414C2, U‐251MG, and PBS groups were sacrificed one hour after administration of [^18^F] FEDAC. The ratios of radioactivity of [^18^F] FEDAC between the ipsilateral and contralateral sides, calculated as ipsilateral‐to‐contralateral ratio (ICR) (Figure 5B), were 2.7 ± 1.2 for the 253G1 group, 1.4 ± 0.2 for the 414C2 group, 4.0 ± 0.8 for the U‐251MG group, and 1.1 ± 0.1 for the PBS group (*n* = 5, 4, 5, and 4, respectively), and were significantly higher in the 253G1 and U‐251MG groups compared with the PBS group (253G1 group, *P* < .05 and U‐251MG group, *P* < .001).

Furthermore, to examine the effects of [^18^F] FEDAC‐PET on spinal cord models, we conducted PET imaging and autoradiography using mice transplanted with 253G1‐NS/PCs and U‐251MG into the intact spinal cord (C5 or T10 level). Although the PET signal was not sufficiently clear due to spillover effects from neighboring organs (collected but unpublished data), ex vivo autoradiography with [^18^F] FEDAC successfully detected radioactive accumulation corresponding to the transplanted area of the spinal cord in the 253G1 and U‐251MG groups (Figure S2).

### 3.5 The intensity of [^18^F] FEDAC‐signal depended on the levels of Nestin and TSPO expression in 253G1‐NS/PCs‐derived remnant immature neural tissues

**Figure 6:**
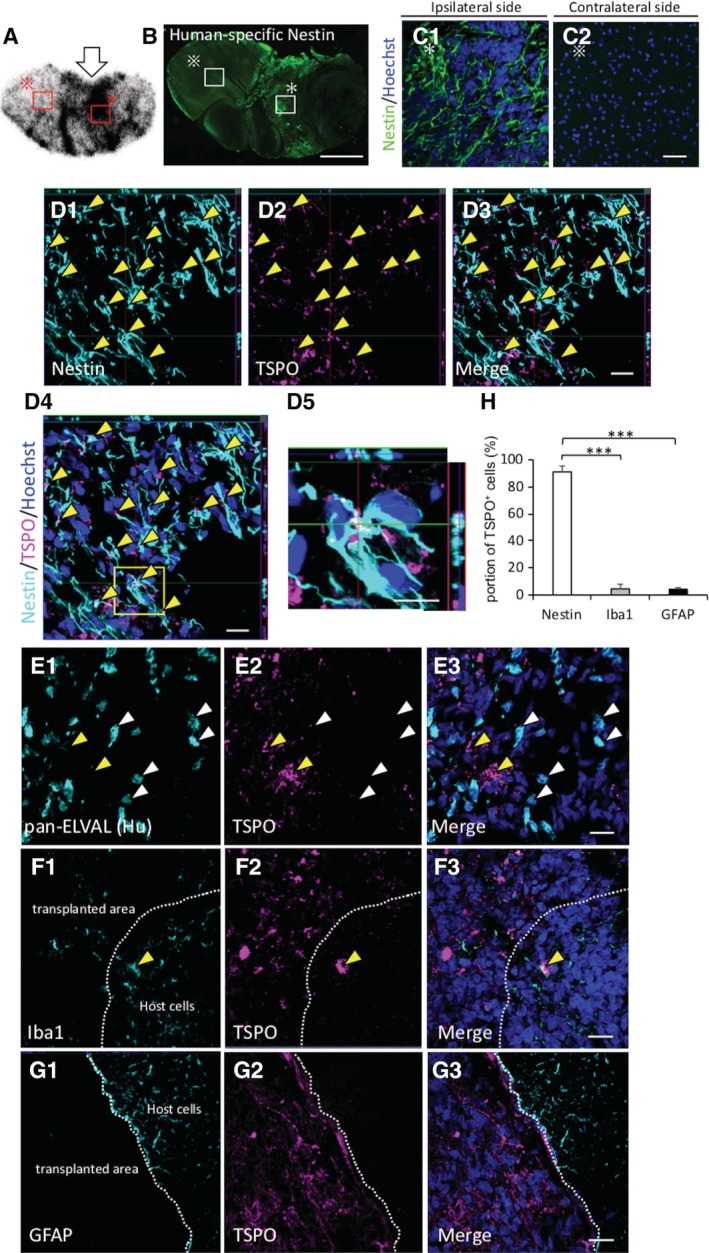
The high uptake of [^18^F] FEDAC in the 253G1‐NS/PCs‐grafted area exclusively comprised TSPO^+^ and Nestin^+^ cells. A‐C, Representative coronal images of 253G1‐NS/PCs‐grafted mouse brain sections in ex vivo autoradiography with [^18^F] FEDAC and immunostained with Nestin eight weeks post‐transplantation (white arrowheads indicate the 253G1‐NS/PCs transplanted site). Representative images of immunohistochemical staining for each cell‐specific type markers. TSPO/Nestin (yellow arrowheads indicate TSPO and Nestin colocalized cells) (D); TSPO/pan‐ELAVL (Hu) (a human‐specific neuron marker) (white arrowheads indicate TSPO^+^ and Hu^−^ cells; yellow arrowheads indicate TSPO^−^ and Hu^+^ neurons) (E); TSPO/Iba1 (microglia) (yellow arrowheads indicate negligible amounts of host cells‐derived TSPO^+^ and Iba1^+^ microglia) (F); TSPO/GFAP (astrocyte) (G). H, Bar graph showing the percentage of TSPO^+^ cells for each cell‐specific marker; Nestin, Iba1, and GFAP. The nuclei were stained with Hoechst 33258. Scale bars = 1000 μm in (B), 20 μm in (B‐G). Values are mean ± SD (n = 5). ***P < .001. GFAP, glial fibrillary acidic protein; NS/PCs, neural stem/progenitor cells

To assess the histological profile of the hiPSC‐NS/PCs‐grafted brain tissues at eight weeks post‐transplantation and to determine the cellular source of radioactive signal derived from [^18^F] FEDAC, we performed immunostaining with TSPO and each cell‐type‐specific marker on sections corresponding to those tested with ex vivo autoradiography (Figure 6). We labeled graft cells with Venus fluorescent protein engineered from the original GFP^32^ and STEM121 (cytoplasm in human cell) (ie, human) (Figure S3). The area showing strong radioactivity on the [^18^F] FEDAC‐ autoradiography was composed of largely Nestin^+^ cells (Figure 6A‐C). In particular, these lesions were mainly co‐localized with TSPO (Figure 6D). In contrast, pan‐ELAVL (Hu)^+^ neurons did not show TSPO expression (Figure 6E). Furthermore, considering the influence of inflammatory cells such as microglia/macrophages^18^, ^33^ and reactive astrocytes^21^ to the [^18^F] FEDAC signal, sections were stained with Iba1 (microglia/macrophage) and GFAP (astrocytes) (Figure 6F,G). According to a quantitative analysis of the immunohistochemical data (Figure 6H), TSPO^+^ cells were highly colocalized with Nestin (91.1% ± 0.4%) rather than Iba1 (4.5% ± 1.2%) (*P* < .001) and GFAP (4.2% ± 3.4) (*P* < .001). The results indicated that a few Iba1^+^ and GFAP^+^ cells were present within the graft area but only a small number of those cells minimally expressed TSPO. All together, we observed that transplanting the 253G1‐NS/PCs could generate poorly differentiated neural tissues at the 8 week point, post‐transplantation, and these immature Nestin^+^ cells preferentially contributed to the development of TSPO expression in hiPSC‐NS/PCs‐grafted mice. Therefore, the radioactive signal derived from [^18^F] FEDAC in the 253G1 group was relatively dependent on TSPO in Nestin^+^ cells. Similar results were observed in the intact spinal cord models in the U‐251MG and 253G1 groups (Figure S4).

## 4 DISCUSSION

The current results revealed that the TSPO protein was expressed in hiPSC‐NS/PCs, and its density decreased as neuronal differentiation progressed in vitro. In contrast, undifferentiated hiPSC‐NS/PCs‐derived cells maintained high levels of TSPO expression. In the in vivo study, [^18^F] FEDAC‐PET showed radioactive accumulation in the transplanted site following 253G1‐NS/PCs transplantation, anatomically validated by ex vivo autoradiography. Immunohistochemical analysis confirmed immature Nestin^+^ neural cells also express TSPO. Collectively, these results show that PET with [^18^F] FEDAC can detect undifferentiated cells following hiPSC‐NS/PCs transplantation into the brain of NOD‐SCID mice, taking advantage of the tendency of less‐mature cells to preferentially express TSPO.

TSPO facilitates diverse cellular functions, including mitochondrial respiration, cholesterol transport, cell proliferation, and apoptosis.^34^ TSPO also plays a crucial role in neural development.^35^ In addition, TSPO is expressed in NS/PCs but not in mature neurons.^22^ The present study confirmed TSPO expression in hiPSC‐NS/PCs which is similar to mouse neuroectodermal stem cells. Particularly, poorly differentiated hiPSC‐NS/PCs‐derived neural cells (ie, 253G1‐d14) exhibited significantly higher levels of TSPO compared to well‐differentiated hiPSC‐NS/PCs‐derived neurons (ie, 414C2‐d14). Immunocytochemical analyses revealed that β III tubulin^+^ neurons derived from hiPSC‐NS/PCs exhibited decreased TSPO levels, while immature Nestin^+^ cells still expressed high levels of TSPO through neuronal induction (Figure 1). As a reference to the analysis described in this study, it is important to accurately note the expression of TSPO in hiPSC‐NS/PCs‐derived neurons. In previous studies, the expression of TSPO is lost in mature neurons but immature neurons express a very low level of TSPO.^16^, ^22^ In the present study, immature β III tubulin^+^ hiPSC‐NS/PCs‐derived neurons seemed to express little amounts of TSPO (Figure 1C,E). In order to elucidate the TSPO expression status in hiPSC‐NS/PC‐derived mature neurons, we performed immunostaining with NeuN (a mature neuronal marker) and TSPO on the 414C2‐d14, and observed complete downregulation of TSPO expression in NeuN^+^ mature hiPSC‐NS/PCs‐derived neurons similar to previous reports using mouse neuroectodermal stem cells^22^ (Figure 1D). The expression pattern of TSPO was consistent with the results of RT‐PCR and Western blot analyses (Figure 2). These results indicated significantly higher neuronal differentiation in 414C2‐NS/PCs relative to 253G1‐NS/PCs, and the extent of differentiation was proportional to the degree of TSPO downregulation.

Although [^11^C]PK11195 is the most frequently used TSPO ligand for PET imaging,^35^, ^36^ its high level of nonspecific binding is reported to result in a low target‐to‐background contrast.^37^ In contrast, [^18^F] FEDAC has higher specificity to TSPO, improving the sensitivity of PET imaging.^19^, ^38^ Studies on [^18^F] FEDAC‐PET have already reported its efficacy in the detection of CNS inflammation in neurodegenerative diseases and cerebral infarction in animal models.^19^ [^18^F] FEDAC is also more convenient and useful because of its relatively longer half‐life (^18^F: 110 minutes, ^11^C: 20 minutes), which reduces the dependency on an on‐site cyclotron when reaching other facilities. Several PET studies using [^18^F] FEDAC in rodent models have shown in vivo specific binding with TSPO and high radioactive signals in TSPO‐rich organs.^19^, ^39^ In contrast, the uptake of [^18^F] FEDAC in healthy CNS is reported to be much lower.^40^ Therefore, PET with [^18^F] FEDAC has the potential to detect early CNS disorders related to TSPO. In this study, PET with [^18^F] FEDAC clearly detected radioactive accumulation in the graft area of the mouse brain in only the 253G1 and U‐251MG (positive control) groups. Four out of five mice in the 414C2 group were used for analysis (one mouse died before detection), and none of the mice exhibited any significant radioactive uptake (Figure 3A). However, small‐animal PET, especially rodent models, has limitations in concisely evaluating the radial uptake due to poor resolution.^41^ Therefore, we put more emphasis on the comparison between the ipsilateral side and contralateral side, and ex vivo autoradiography to validate the accumulation of the tracer. Accordingly, the PET data revealed that the AUC values on the ipsilateral side of the brain (ie, the transplanted site) in the 253G1 and U‐251MG groups were significantly higher than those on the contralateral side of the brain (ie, the intact side) (Figure 3B). Furthermore, ex vivo autoradiography confirmed that the anatomical distribution of radioactive accumulation on the transplanted site (Figure 5A) and the ICR were significantly higher in the 253G1 group compared to the PBS‐injected group (Figure 5B). On the other hand, there was no significant difference between the 253G1 group (2.7 ± 1.2, n = 5) and the 414C2 group (1.4 ± 0.2, n = 4) in the ICR, despite the relatively higher tendency for radioactive accumulation in the 235G1 group. In general, autoradiography offers an order of magnitude higher spatial resolution than PET.^42^ Therefore, ex vivo autoradiography was also able to detect radial accumulation of residual immature tissues in the 414C2 group two months post‐transplantation (Figure S5). This observation is not unexpected since non‐proliferative undifferentiated cells may persist for several months (2, 3) post‐transplantation even when non‐tumorigenic hiPSC‐NS/PCs are used.^5^, ^9^ In other words, device development associated with PET, such as higher resolution and sensitivity PET camera system or better tracer, would be needed to detect low numbers of undifferentiated cells in the future. In addition, we observed [^18^F] FEDAC radioactivity in the intact spinal cords of 253G1‐NS/PCs and U‐251MG transplanted mice in an ex vivo autoradiography study (Figure S2). These results suggest that PET with [^18^F] FEDAC could provide a suitable imaging tool for the diagnosis of CNS diseases associated with TSPO elevation in clinical settings.

Due to the tendency of different cell types to express TSPO, it was necessary to determine which cells were responsible for the increased radioactive uptake, since we transplanted NS/PCs that were initially composed of heterogeneous neural cells. A large number of previous studies with radiolabeled TSPO ligands reported that cellular localization of elevated TSPO expression was exclusively confined to activated microglia or reactive astrocytes.^18^, ^21^, ^43^ Therefore, we performed immunohistochemical analysis on sections corresponding to those used in ex vivo autoradiography. The 253G1‐NS/PCs‐derived tissues were consistently comprised of Nestin^+^ immature cells expressing TSPO (Figure 6A‐D). A few Iba1^+^ microglia and GFAP^+^ astrocytes were observed within the graft area, but only partially contributed to the overall TSPO expression (Figure 6G,H). It is likely that these results reflect a similar pattern to the TSPO expression of glioma‐associated microglia/macrophages (GAMs).^13^, ^44^, ^45^ A previous report demonstrated that GAMs contributed to the uptake in glioma PET imaging with the TSPO radioligand, but their influence on glioma TSPO expression was weak.^46^ Together, these results suggested that the detected signal by PET with [^18^F] FEDAC would be predominantly derived from Nestin^+^ undifferentiated neural cells after hiPSC‐NS/PCs transplantation, and not microglia nor astrocytes associated with neuroinflammation. Additionally, elevated levels of TSPO in inflammatory cells in response to lesions are reported to be directly related to the extent of the damage.^47^, ^48^ In the present study, we utilized these intact models after enough time had passed post‐transplantation to exclude inflammatory factors that may have contributed to the total TSPO signal.^33^, ^49^, ^50^

In order to validate the clinical application of [^18^F] FEDAC‐PET, we performed histological analysis to determine the levels of TSPO expression and the percentage of TSPO^+^ cells in SCI mice 103 days post hiPSC‐NS/PCs transplantation (Figure S6). We observed robust Nestin^+^ and TSPO^+^ undifferentiated tissues in the injured spinal cord as well as the intact spinal cord (Figure S4), whereas Iba1^+^ cells and GFAP^+^ cells in the injured spinal cord expressed negligible amounts of TSPO. These results suggest that [^18^F] FEDAC‐PET is a useful diagnostic tool which can preferentially detect neoplastic changes due to the proliferation of TSPO^+^ undifferentiated cells in the injured spinal cord.

A range of human stem cell‐based therapy methods have been developed for clinical applications in the treatment of ischemic stroke and CNS injury.^51^, ^52^, ^53^ Several noninvasive methods are safe for evaluation of cell‐tracking. MRI, as a representative imaging modality, provides anatomical details of target organs with a high level of clinical accessibility.^54^ We observed that the gadolinium enhanced areas corresponded with high tracer uptake in [^18^F] FEDAC‐PET (Figure 4). However, MRI data have a lack of a molecular targeting capability of the gadolinium chelate contrast agent. In contrast, PET can detect faint metabolic processes and provide quantitative data useful for the early detection of lesions.^55^

In clinical application of patients with chronic TBI or SCI, we believe selection of the timing of the PET scan is essential in diagnosing tumor‐like overgrowth of remnant undifferentiated hiPSC‐NS/PCs by monitoring the changes in the total amount of TSPO. The main focus of our present study on [^18^F] FEDAC‐PET was not to selectively identify TSPO‐expressing cells, but to monitor the total amount of the TSPO signal post hiPSC‐NS/PCs transplantation and to detect tumorigenesis of the grafted tissues as early as possible. Information on the elevation of TSPO would be useful in detecting tumorigenicity of the transplanted hiPSC‐NS/PCs by carefully selecting when to perform the PET scan. For example, there is a dramatic increase in inflammation and associated activation of TSPO‐expressing microglia after TBI or SCI.^33^, ^56^ Moreover, it has been reported that the activated Iba1^+^ microglia, which present a major cellular source of TSPO, peaks 42 days post‐SCI^50^ while TSPO expression peaks within a week or 72 hours after infarction, especially in the cerebral infarction models and the brain injury models, respectively.^56^, ^57^ In order to elucidate time‐dependent changes of TSPO expression after SCI in rodent models, we analyzed the gene expression of TSPO in mice at the nine days marker and 42 days marker after SCI using microarray. We observed that TSPO was significantly elevated 9 days after injury (dpi) (Figure S7) and there was no significant difference between 9 and 42 dpi. Accordingly, we showed that the TSPO signal associated with inflammation reached its peak within 6 weeks post‐TBI or SCI in rodent models. In the clinical setting, transplantation is routinely performed on TBI or SCI patients in the chronic phase, at which point inflammation should have significantly subsided. Therefore, TSPO expression, including the inflammation‐associated one is expected to be significantly downregulated in chronic patients. On the other hand, there is a transient elevation of TSPO at the point of hiPSC‐NS/PCs transplantation. However, if the transplanted hiPSC‐NS/PCs are nontumorigenic, they would undergo the differentiation into neurons and glia appropriately and TSPO expression, accordingly, would be downregulated over time. In contrast, the total TSPO expression will be upregulated as tumorigenic hiPSC‐NS/PCs expand. [^18^F] FEDAC‐PET would therefore preferentially detect high levels of TSPO expression by tumorigenic transplanted hiPSC‐NS/PCs in the absence of primary inflammation.

In cases where possible tumorigenic transformation is detected using [^18^F] FEDAC‐PET, we are suggesting some counter measures to tumorigenicity post‐NS/PCs transplantation. First, discontinuing the immunosuppressant drugs to ablate graft cells is a hopeful approach against the formation of tumors.^24^ Second, the use of suicide gene therapy in ablating transplanted hiPSC‐NS/PCs.^58^, ^59^ Third, focal surgical excision in the case of patients with complete paralysis since no further exacerbation of symptoms occur. Accordingly, [^18^F] FEDAC‐PET would be a useful judgment tool for taking the abovementioned control measures with conventional imaging modalities such as MRI and CT when complications accompanied with NS/PCs transplantation occur.

Although the present study validated the efficacy of [^18^F] FEDAC‐PET in intact murine models to visualize residual proliferative undifferentiated hiPSC‐NS/PCs, based on in vitro data, it should be acknowledged that there are several limitations to be resolved. One example is the limited PET resolution of rodent CNS, particularly rodent spinal cords, due to their thickness (1‐2 mm), and signal spillover by TSPO‐rich organs which made it difficult to correctly evaluate radioactive uptake. This intrinsic limitation is generally associated with rodent models. ex vivo autoradiography with [^18^F] FEDAC however confirmed high radioactivity in the brain and the spinal cord models. The second limitation was the inability to determine the time of emergence of defective less‐mature neural cells and the limit of detectability in clinical settings. This would have required taking PET scans at different time points in the same clinical‐grade hiPSC‐NS/PCs‐bearing large animal model such as a nonhuman primate to overcome the limited resolution. Third, although [^18^F] FEDAC‐PET cannot distinguish between activated glial cells and undifferentiated NS/PCs as cellular sources of TSPO as mentioned above, the main focus of this study was to monitor tumorigenic changes of grafted hiPSC‐NS/PCs by capturing the elevated TSPO signal.

This study is the first report of [^18^F] FEDAC‐PET as a tool for visualizing remnant proliferative immature cells after the transplantation of hiPSC‐NS/PCs in intact mouse models when the effect of inflammation on TSPO expression is not significant. Although the [^18^F] FEDAC‐PET signal change is a time dependent, it can be used in conjunction with conventional modalities, such as MRI and CT, to visualize tumorigenicity of transplanted cells.

## 5 CONCLUSION

We confirmed TSPO expression in hiPSC‐NS/PCs in vitro, and the efficiency of PET with [^18^F] FEDAC to visualize less‐mature cells following hiPSC‐NS/PCs transplantation into mouse brains in vivo. We hope that this imaging technique would contribute to the safety of stem cell‐based transplantation therapy for neurotrauma.

## CONFLICT OF INTEREST

Y.F. declared leadership position with CTO, CMI Inc. M.N. declared consultancy role with K‐Pharma and research funding from RMic, Hisamitsu. H.O. declared leadership position at Keio University School of Medicine and is a compensated scientific consultant for San Bio Co. Ltd. and K Pharma Inc. The other authors declared no potential conflicts of interest.

## AUTHOR CONTRIBUTIONS

Y.T.: designed the project, performed experiments, interpreted the data, wrote the manuscript; T.Y., N.N., Y.N., S.N., S.N., T.I., M.O., O.T., B.J., I.A., M.J., M.M., Y.F.: technical assistance; M.Z.: provided experimental support, ideas for the project; M.N., H.O.: designed the studies, supervised the overall project, prepared the final manuscript.

## Supporting information


**Fig. S1 Bioluminescence tracking of the transplanted hiPSC‐NS/PCs. (A)**: Quantitative analyses of the photon counts derived from the grafted cells for four weeks (the control U‐251MG group was evaluated for two weeks due to their survival period). The grafted 253G‐NS/PCs proliferated more rapidly than that of the 414C2‐NS/PCs. Abbreviations: hiPSC‐NS/PCs, human induced pluripotent stem cells derived neural stem/progenitor cells.

**Fig. S2 *Ex vivo* autoradiography with [** ^**18**^ **F] FEDAC in the 253G1‐NS/PCs‐grafted mouse spinal cords. (A‐D)**: Representative autoradiography images of the 253G1‐NS/PCs, U‐251MG or PBS grafted mouse spinal cords after injection of the [^18^F] FEDAC. C5 level of the spinal cord grafted with 253G1‐NS/PCs **(A)**; and U‐251MG **(B)**; T10 level of the spinal cord grafted with U‐251MG **(C)**; and PBS **(D)**. Arrows indicate the transplanted site. Abbreviations: NS/PCs, neural stem/progenitor cells; PBS, phosphate‐buffered saline.

**Fig. S3 Histological analyses of the 253G1‐NS/PCs‐grafted mouse brains (related to Figure 6). (A)**: Representative hematoxylin and eosin image of the coronal section 56 days post‐transplantation. The grafted cells were labeled with Venus/GFP/anti‐human cytoplasm (STEM121)^+^ and TSPO/Nestin **(B)**; TSPO/pan‐ELAVL (Hu) (a human specific neuron marker) **(C)**; TSPO/Iba1 (microglia) **(D)**; TSPO/GFAP (astrocyte) **(E)**. The nuclei were stained with Hoechst 33258. Scale bars, 1000 μm in (A), 20 μm in **(B‐E)**. Abbreviations: NS/PCs, neural stem/progenitor cells; GFP, green fluorescent protein; GFAP, glial fibrillary acidic protein.

**Fig. S4 Histological analyses of the U‐251MG‐ or 253G1‐NS/PCs‐grafted intact spinal cords of NOD/SCID mice. (A)** Representative hematoxylin and eosin (H &E) sagittal image of the U‐251MG‐grafted spinal cord section 21 days post transplantation. **(B)** Representative image of the U‐251MG‐ grafted spinal cord section immunostained with Venus/GFP, TSPO and Nestin. **(C)** Representative H &E sagittal image of the 253G1‐NS/PCs‐grafted spinal cord sections 56 days post‐transplantation. **(D)** Representative image of the 253G1‐NS/PCs‐grafted spinal cord section immunostained with Venus/GFP, TSPO and Nestin. The nuclei were stained with Hoechst 33258. Scale bars, 1000 μm in **(A and C)**, 20 μm in **(B and D)**. Abbreviations: NS/PCs, neural stem/progenitor cells; GFP, green fluorescent protein.

**Fig. S5 *Ex vivo* autoradiography with [** ^**18**^ **F] FEDAC and immunohistological analyses on the 414C2‐NS/PCs‐grafted brain of NOD/SCID mice. (A)**: Representative coronal images of 414C2‐NS/PCs‐grafted brain sections in *ex vivo* autoradiography with [^18^F] FEDAC (red box indicates the graft area). **(B)**: Magnified regions are indicated by the yellow box, showing representative coronal images of the 414C2‐NS/PCs‐grafted mouse brain sections immunostained with Venus/GFP, TSPO and Nestin. The nuclei were stained with Hoechst 33258. Scale bars, 1000 μm in (A), 20 μm in **(B)**. Abbreviations: NS/PCs, neural stem/progenitor cells; GFP, green fluorescent protein.

**Fig. S6 TSPO expression in immature neural cells in 253G1‐NS/PCs‐grafted injured spinal cord of NOD/SCID mice (103 days post transplantation).** In the present study, TSPO immunostaining was newly performed for specimen derived from 253G1‐NS/PCs‐grafted NOD/SCID mice SCI models, which were generated in our previous studies^3^. **(A)**: Representative hematoxylin and eosin sagittal image of 253G1‐NS/PCs‐grafted inured spinal cord. **(B‐D)**: Representative images of immunohistochemical staining for each cell‐specific type markers. TSPO/Nestin (yellow arrowheads indicate TSPO^+^/Nestin^+^ cells while white arrow heads indicate TSPO^+^/Nestin^−^ cells; TSPO/Iba1 (microglia) **(C)**; TSPO/GFAP (astrocyte) (yellow arrowheads indicate TSPO^+^/GFAP^−^ cells while white arrow heads indicate TSPO^+^/GFAP^+^ cells **(D)**. **(E)**: Bar graph showing the percentage of TSPO^+^ cells for each cell‐specific marker; Nestin, Iba1 and GFAP. The nuclei were stained with Hoechst 33258. Scale bars, 1000 μm in (A), 20 μm in **(B‐D)**. Values are means ± SD (n = 3). ***P < 0.001 according to one‐way ANOVA with the Tukey‐Kramer test. Abbreviations: NS/PCs, neural stem/progenitor cells; GFAP, glial fibrillary acidic protein.

**Fig. S7 Temporal changes of *TSPO* mRNA expression after the SCI (9 dpi and 42 dpi)by the re‐analysis of microarray data** ^**1**^ **. Microarray analysis revealed that *TSPO* mRNA expression reached its peak within six weeks post‐SCI in mouse models. (A)**: The microarray data revealed the gene expression signals of *TSPO* at 9 dpi and 42 dpi groups compared with the intact group (equal to 1). *TSPO* mRNA was significantly up‐regulated at 9 dpi and there was no significant difference between 9 dpi and 42 dpi. The data shows the mean fold‐change values vs intact samples. Values are means ± SD (n = 3). *P < 0.05 and not significant (N.S.) according to one‐way ANOVA with the Tukey‐Kramer test. Abbreviations: SCI, spinal cord injury; dpi, day after spinal cord injury.

**Table. S1 (related to Figure 3). DVR of the grafted area in each group** (253G1 group, n = 5, 414C2 group n = 4, U251MG group, n = 5 and PBS group, n = 4). Abbreviations: DVR, distribution volume ratio

**Data S1:** Supporting information

## Data Availability

The data that support the findings of this study are available on request from the corresponding authors.
