# Supplementary figures and images for: In vivo monitoring of remnant undifferentiated neural cells following human induced pluripotent stem cell‐derived neural stem/progenitor cells transplantation
Source: Stem Cells Transl Med. 2020 Jan 6;9(4):465–77. doi: 10.1002/sctm.19-0150 (PMC7103627; doi:10.1002/sctm.19-0150)

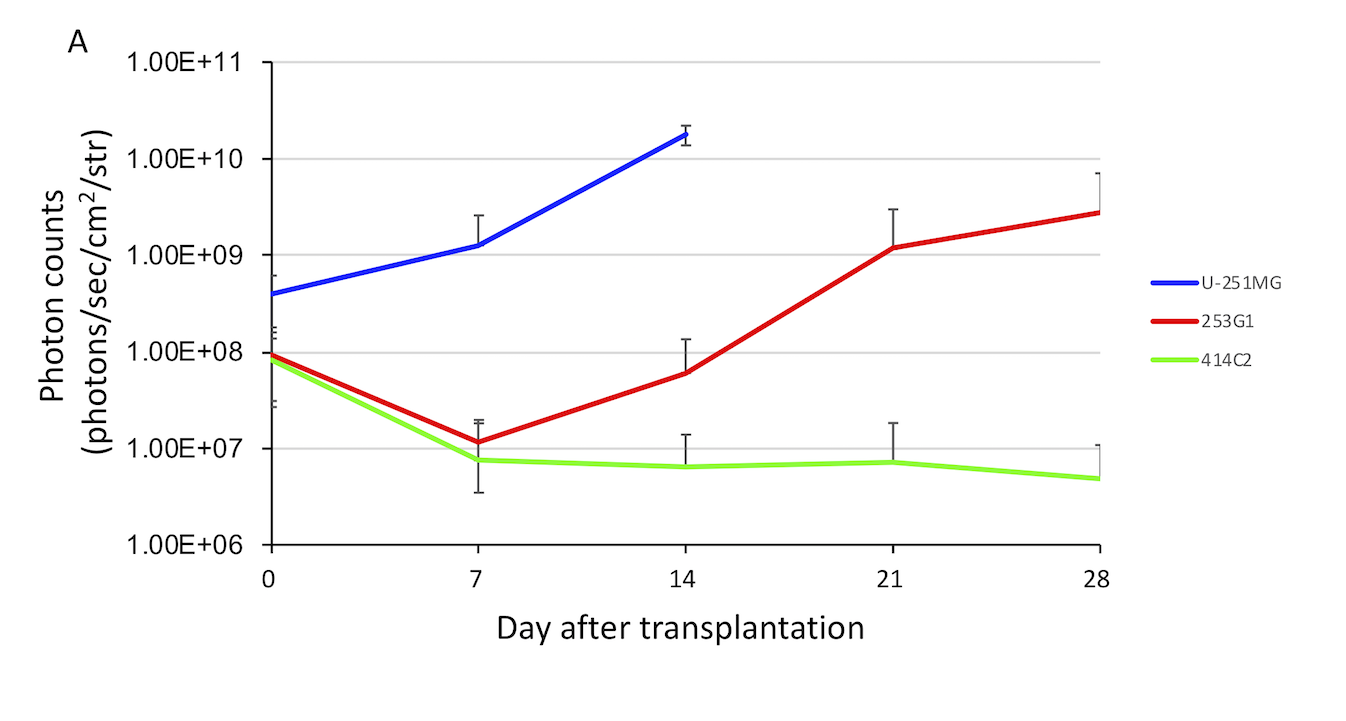

Supplement: Supplementary file 1 — Fig. S1 Bioluminescence tracking of the transplanted hiPSC‐NS/PCs. (A): Quantitative analyses of the photon counts derived from the grafted cells for four weeks (the control U‐251MG group was evaluated for two weeks due to their survival period). The grafted 253G‐NS/PCs proliferated more rapidly than that of the 414C2‐NS/PCs. Abbreviations: hiPSC‐NS/PCs, human induced pluripotent stem cells derived neural stem/progenitor cells. [file SCT3-9-465-s001.tiff]

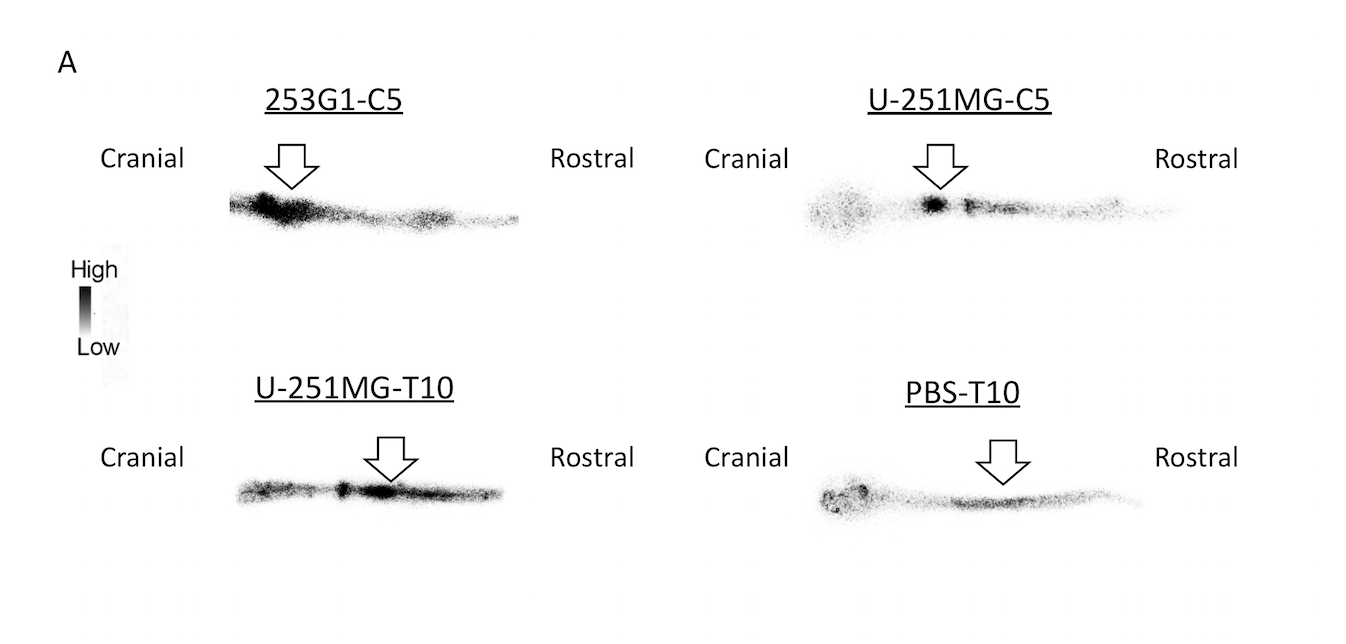

Supplement: Supplementary file 2 — Fig. S2 Ex vivo autoradiography with [ 18 F] FEDAC in the 253G1‐NS/PCs‐grafted mouse spinal cords. (A‐D): Representative autoradiography images of the 253G1‐NS/PCs, U‐251MG or PBS grafted mouse spinal cords after injection of the [18F] FEDAC. C5 level of the spinal cord grafted with 253G1‐NS/PCs (A); and U‐251MG (B); T10 level of the spinal cord grafted with U‐251MG (C); and PBS (D). Arrows indicate the transplanted site. Abbreviations: NS/PCs, neural stem/progenitor cells; PBS, phosphate‐buffered saline. [file SCT3-9-465-s004.tiff]

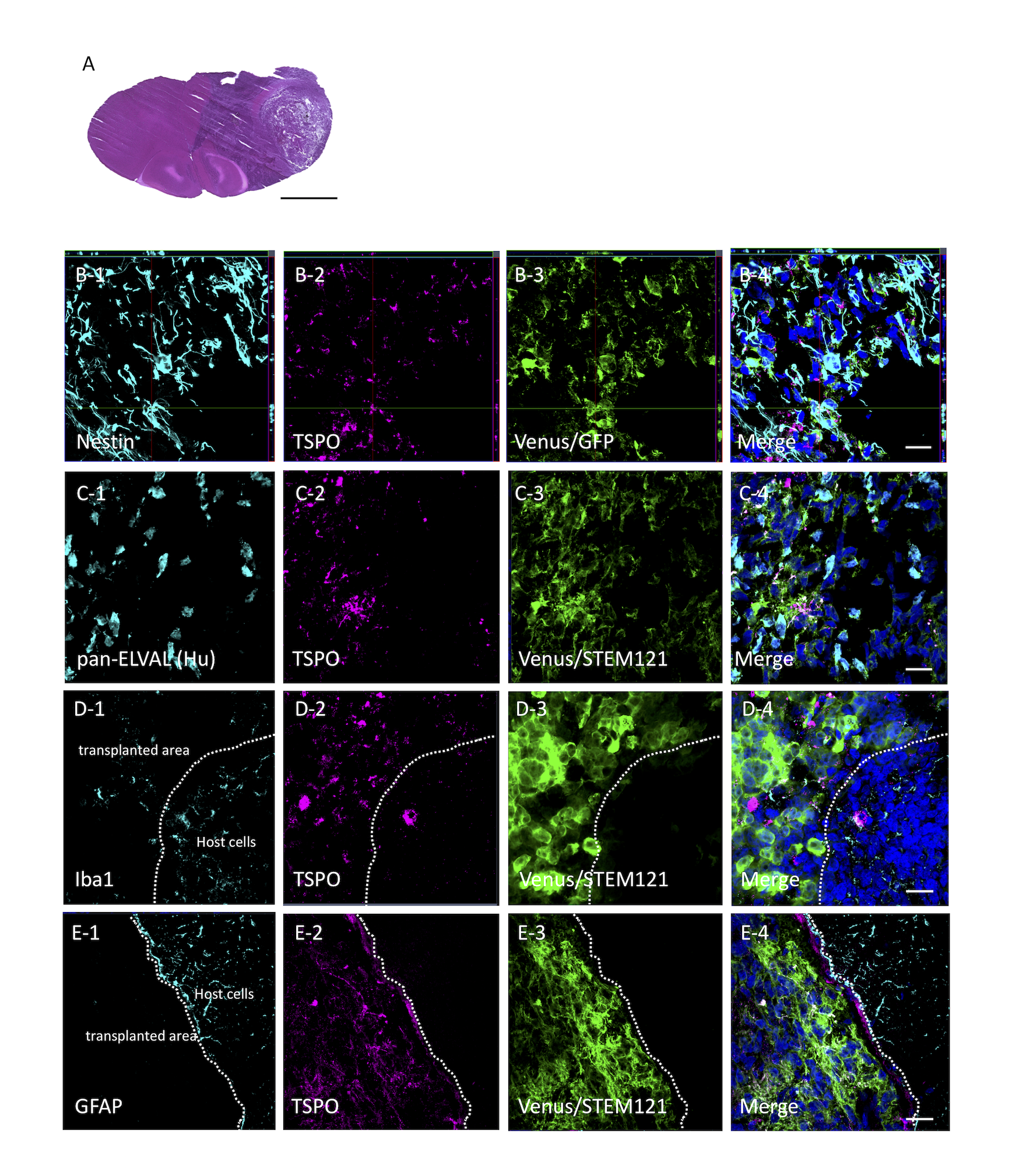

Supplement: Supplementary file 3 — Fig. S3 Histological analyses of the 253G1‐NS/PCs‐grafted mouse brains (related to Figure 6). (A): Representative hematoxylin and eosin image of the coronal section 56 days post‐transplantation. The grafted cells were labeled with Venus/GFP/anti‐human cytoplasm (STEM121)+ and TSPO/Nestin (B); TSPO/pan‐ELAVL (Hu) (a human specific neuron marker) (C); TSPO/Iba1 (microglia) (D); TSPO/GFAP (astrocyte) (E). The nuclei were stained with Hoechst 33258. Scale bars, 1000 μm in (A), 20 μm in (B‐E). Abbreviations: NS/PCs, neural stem/progenitor cells; GFP, green fluorescent protein; GFAP, glial fibrillary acidic protein. [file SCT3-9-465-s005.tiff]

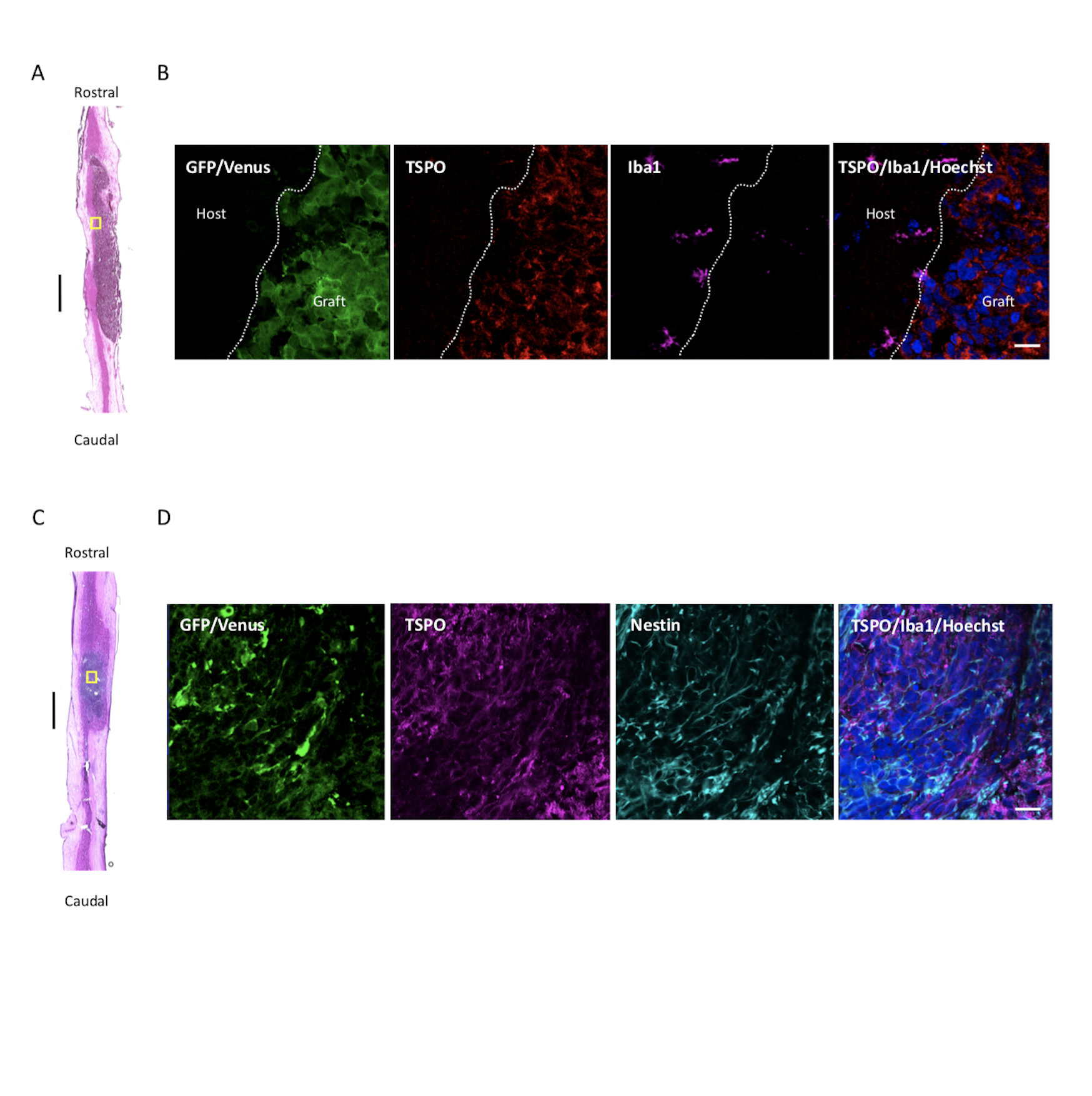

Supplement: Supplementary file 4 — Fig. S4 Histological analyses of the U‐251MG‐ or 253G1‐NS/PCs‐grafted intact spinal cords of NOD/SCID mice. (A) Representative hematoxylin and eosin (H &E) sagittal image of the U‐251MG‐grafted spinal cord section 21 days post transplantation. (B) Representative image of the U‐251MG‐ grafted spinal cord section immunostained with Venus/GFP, TSPO and Nestin. (C) Representative H &E sagittal image of the 253G1‐NS/PCs‐grafted spinal cord sections 56 days post‐transplantation. (D) Representative image of the 253G1‐NS/PCs‐grafted spinal cord section immunostained with Venus/GFP, TSPO and Nestin. The nuclei were stained with Hoechst 33258. Scale bars, 1000 μm in (A and C), 20 μm in (B and D). Abbreviations: NS/PCs, neural stem/progenitor cells; GFP, green fluorescent protein. [file SCT3-9-465-s006.tiff]

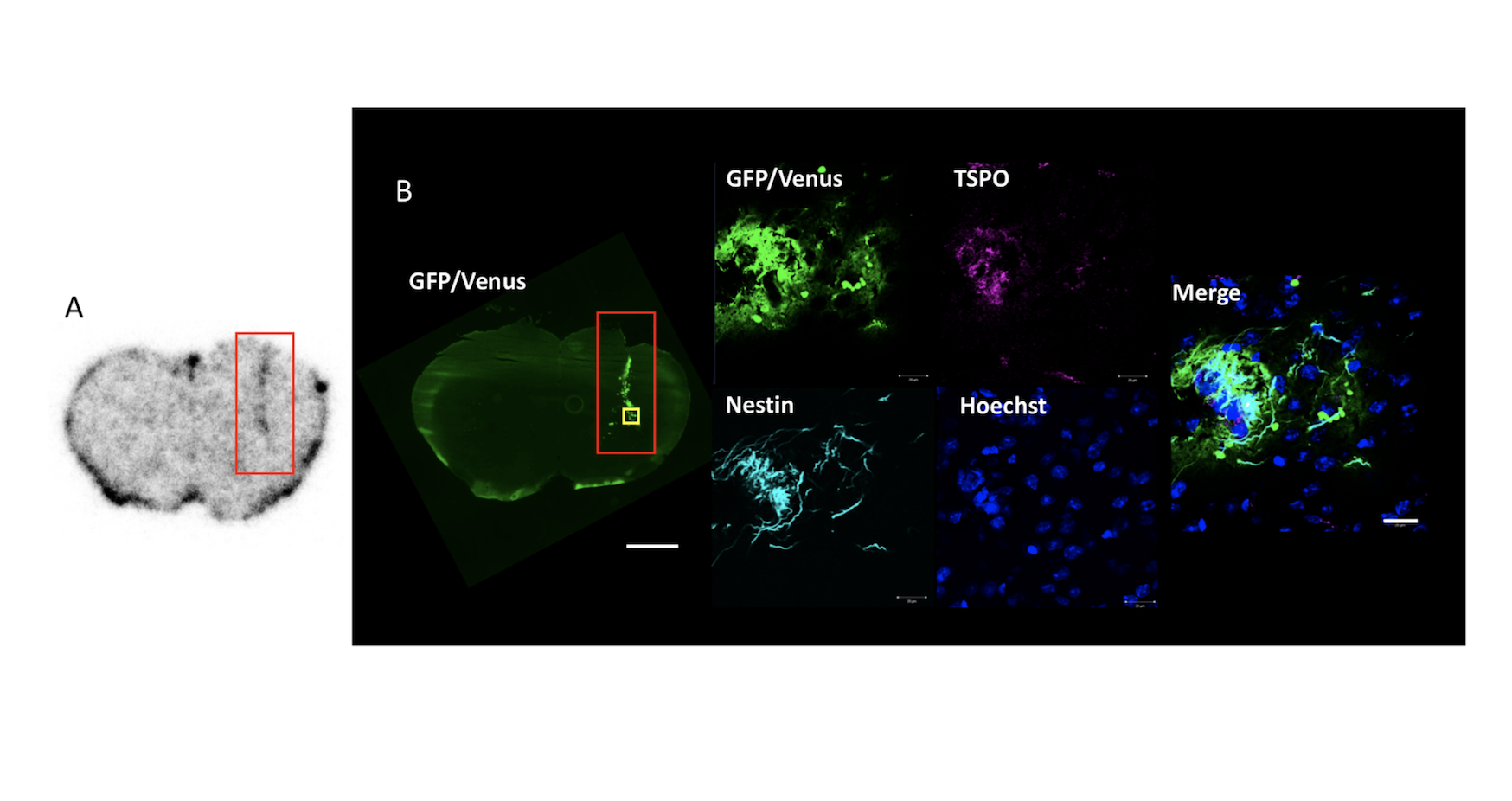

Supplement: Supplementary file 5 — Fig. S5 Ex vivo autoradiography with [ 18 F] FEDAC and immunohistological analyses on the 414C2‐NS/PCs‐grafted brain of NOD/SCID mice. (A): Representative coronal images of 414C2‐NS/PCs‐grafted brain sections in ex vivo autoradiography with [18F] FEDAC (red box indicates the graft area). (B): Magnified regions are indicated by the yellow box, showing representative coronal images of the 414C2‐NS/PCs‐grafted mouse brain sections immunostained with Venus/GFP, TSPO and Nestin. The nuclei were stained with Hoechst 33258. Scale bars, 1000 μm in (A), 20 μm in (B). Abbreviations: NS/PCs, neural stem/progenitor cells; GFP, green fluorescent protein. [file SCT3-9-465-s007.tiff]

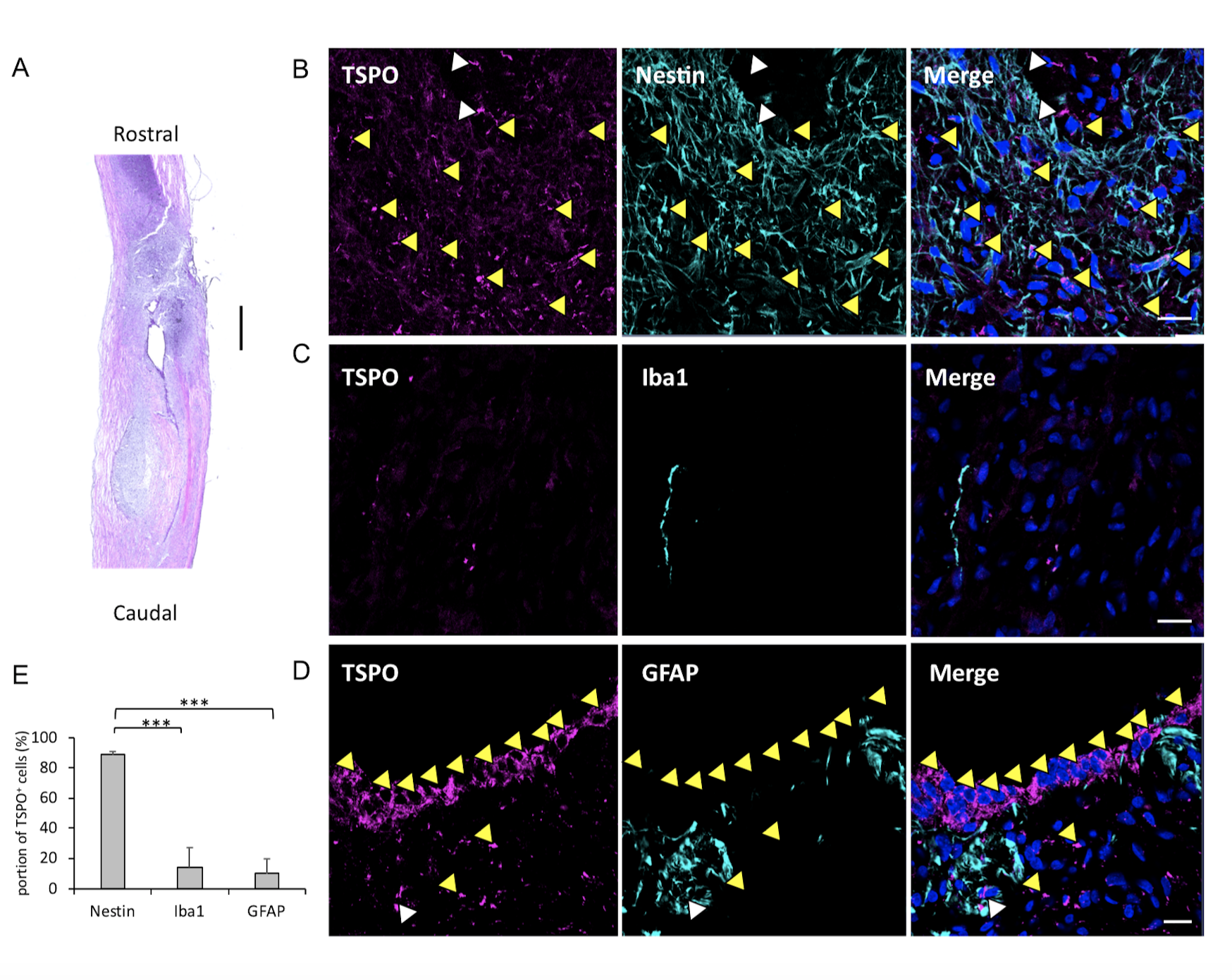

Supplement: Supplementary file 6 — Fig. S6 TSPO expression in immature neural cells in 253G1‐NS/PCs‐grafted injured spinal cord of NOD/SCID mice (103 days post transplantation). In the present study, TSPO immunostaining was newly performed for specimen derived from 253G1‐NS/PCs‐grafted NOD/SCID mice SCI models, which were generated in our previous studies3. (A): Representative hematoxylin and eosin sagittal image of 253G1‐NS/PCs‐grafted inured spinal cord. (B‐D): Representative images of immunohistochemical staining for each cell‐specific type markers. TSPO/Nestin (yellow arrowheads indicate TSPO+/Nestin+ cells while white arrow heads indicate TSPO+/Nestin− cells; TSPO/Iba1 (microglia) (C); TSPO/GFAP (astrocyte) (yellow arrowheads indicate TSPO+/GFAP− cells while white arrow heads indicate TSPO+/GFAP+ cells (D). (E): Bar graph showing the percentage of TSPO+ cells for each cell‐specific marker; Nestin, Iba1 and GFAP. The nuclei were stained with Hoechst 33258. Scale bars, 1000 μm in (A), 20 μm in (B‐D). Values are means ± SD (n = 3). ***P < 0.001 according to one‐way ANOVA with the Tukey‐Kramer test. Abbreviations: NS/PCs, neural stem/progenitor cells; GFAP, glial fibrillary acidic protein. [file SCT3-9-465-s008.tiff]

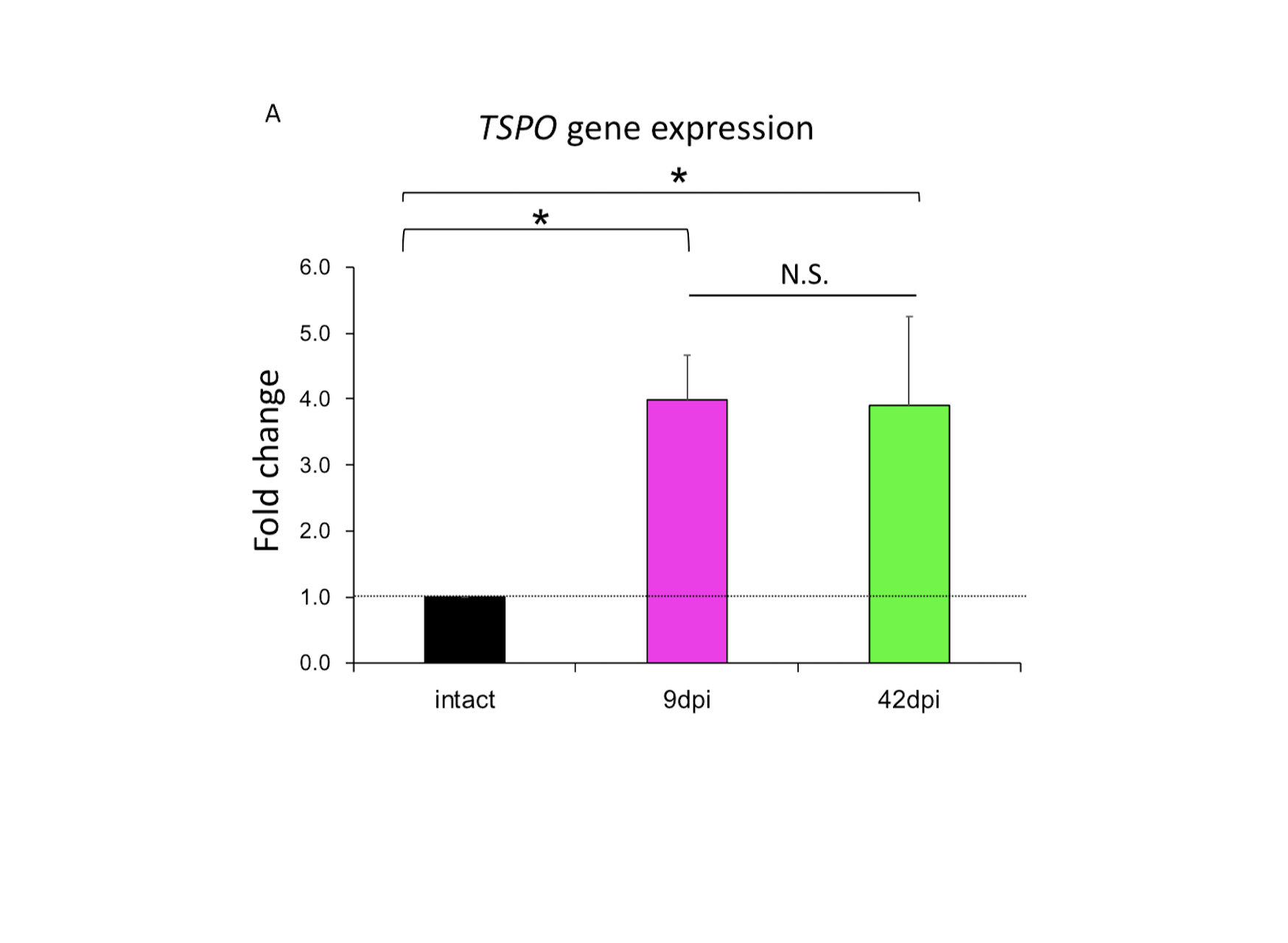

Supplement: Supplementary file 7 — Fig. S7 Temporal changes of TSPO mRNA expression after the SCI (9 dpi and 42 dpi)by the re‐analysis of microarray data 1 . Microarray analysis revealed that TSPO mRNA expression reached its peak within six weeks post‐SCI in mouse models. (A): The microarray data revealed the gene expression signals of TSPO at 9 dpi and 42 dpi groups compared with the intact group (equal to 1). TSPO mRNA was significantly up‐regulated at 9 dpi and there was no significant difference between 9 dpi and 42 dpi. The data shows the mean fold‐change values vs intact samples. Values are means ± SD (n = 3). *P < 0.05 and not significant (N.S.) according to one‐way ANOVA with the Tukey‐Kramer test. Abbreviations: SCI, spinal cord injury; dpi, day after spinal cord injury. [file SCT3-9-465-s009.tiff]

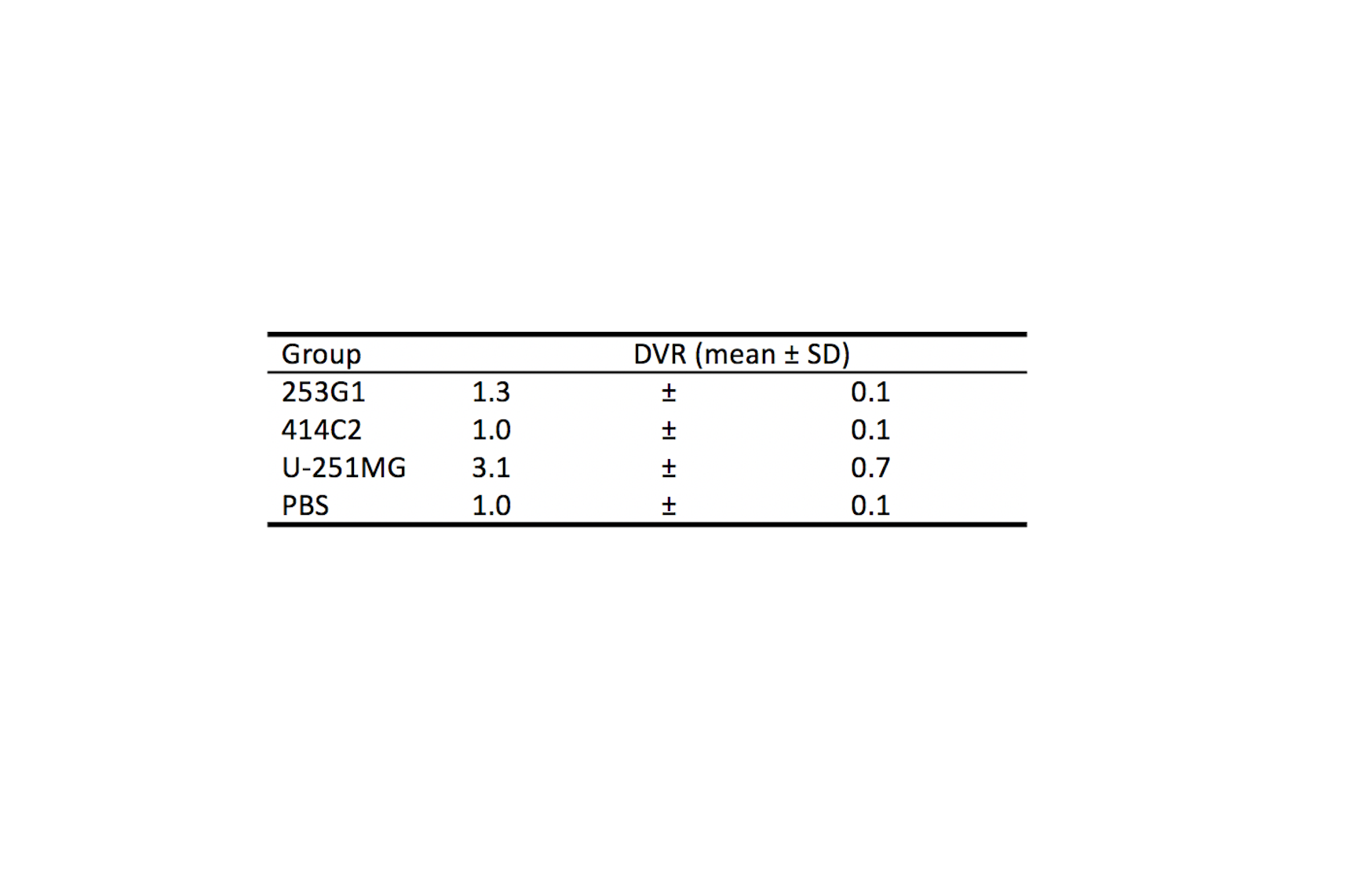

Supplement: Supplementary file 8 — Table. S1 (related to Figure 3). DVR of the grafted area in each group (253G1 group, n = 5, 414C2 group n = 4, U251MG group, n = 5 and PBS group, n = 4). Abbreviations: DVR, distribution volume ratio [file SCT3-9-465-s003.tiff]
