# Supplementary material for: In vivo monitoring of remnant undifferentiated neural cells following human induced pluripotent stem cell‐derived neural stem/progenitor cells transplantation
Source: Stem Cells Transl Med. 2020 Jan 6;9(4):465–77. doi: 10.1002/sctm.19-0150 (PMC7103627; doi:10.1002/sctm.19-0150)
Supplement: Supplementary file 9 — Data S1: Supporting information [file SCT3-9-465-s002.pdf]

Supplementary Information for

In Vivo Monitoring of Remnant Undifferentiated Neural Cells Following Human iPS Cell-Derived Neural Stem/Progenitor Cells Transplantation.

Authors

Yuji Tanimoto, Tomoteru Yamasaki, Narihito Nagoshi, Yuichiro Nishiyama, Satoshi Nori, Soraya Nishimura, Tsuyoshi Iida, Masahiro Ozaki, Osahiko Tsuji, Bin Ji, Ichio Aoki, Masahiro Jinzaki, Morio Matsumoto, Yasuhisa Fujibayashi, Ming-Rong Zhang, *Masaya Nakamura and *Hideyuki Okano

Corresponding Authors

*Masaya Nakamura, Department of Orthopaedic Surgery, Keio University School of Medicine, Tokyo, 160-8582, Japan: masa@keio.jp

*Hideyuki Okano, Department of Physiology, Keio University School of Medicine, Tokyo, 160-8582, Japan, email: hidokano@keio.jp

SI Materials and Methods

RT-PCR

Total RNA was extracted from hiPSC-NS/PCs-derived cells cultured for 14 days and U-251MG (human glioblastoma) using a RNeasy Micro Kit (Qiagen Inc., Hilgen, Germany), and reverse transcribed with the ReverTra Ace qPCR RT aster mix (TOYOBO co., Ltd. Life Science Department, Osaka, Japan). Quantitative polymerase chain reactions (QT-PCR) were carried out using Step One Plus^{TM} (Applied Biosystems, Foster city, CA, USA), according to the manufacturer's instructions. The expression levels of the TSPO gene were normalized to that of GAPDH using the ΔΔCT method. We selected primers used for the reactions from TaqMan gene expression assays and TaqMan gene expression 96-well fast plates (Life Technologies, Carlsbad, CA, USA).

Western Blotting Assay

Total protein was extracted from hiPSC-NS/PCs-derived cells cultured for day 1 and day 14, then homogenized in lysis buffer. 10 μg of proteins were electrophoretically separated on 4%-to-20% gradient polyacrylamide gels and transferred onto polyvinylidene difluoride (PVDF) membranes. Membranes were blocked with Blocking One (Nacalai Tesque, Kyoto, Japan) for 1 hour at room temperature (RT) then incubated overnight at 4 °C with the following primary antibodies: anti-human-specific TSPO (NP157, rabbit IgG, 1:5000; National Institute for Quantum and Radiological Science and Technology, Chiba, Japan), anti-human-specific Nestin protein (MAB5326, mouse IgG1, 1:5000; Merck Millipore, Billerica, Massachusetts, USA), anti-ß III-tubulin (T8660, mouse IgG2b, 1:5000; Sigma-Aldrich, St. Louis, MO, USA). After membranes were washed with Tris-buffered saline, 0.1% Tween 20, they were incubated with horseradish peroxidase-conjugated secondary antibody for 1 hour at RT. The bands were visualized using an enhanced chemiluminescence reagent (GE Healthcare, Buckinghamshire, UK) and the ImageQuant LAS 4000 instrument (GE Healthcare). The quantification of each band was performed using the NIH image analyzer. The ratio of band intensity among TSPO, Nestin, ß III tubulin relative to beta-actin was calculated to correct any variation in the protein gel loading.

Transplantation

Adult female NOD-SCID mice (8-weeks-old, 20–22 g; Clea, Tokyo, Japan) were anesthetized intraperitoneally (i.p.) with ketamine (100 mg/kg) and xylazine (10 mg/kg). Donor cells were cultured *in vitro* from 253G1-NS/PCs, 414C2 -NS/PCs and U-251MG. For the brain models, 5 × 10^5/2 µl of 253G1-NS/PCs, 414C2-NS/PCs and U-251MG (n = 5, for each cell type) were stereotactically transplanted into the right striatum (2 mm lateral and 1 mm anterior to bregma; depth 3 mm from dura) of each mouse with a glass micropipette at a rate of 1 µl/min using a Hamilton syringe (25 µl) and a stereotaxic micro injector (KDS 310; Muromachi Kikai Co. Ltd., Tokyo, Japan). These mice were used for PET, MRI, autoradiography and histological analyses. PBS was used in the negative control group (*n* = 4). For the spinal cord models, 5 × 10^5 / 2 µl of 253G1-NS/PCs or U-251MG were transplanted into intact spinal cords at the level of C5 (*n* = 3, each), and U-251MG and PBS were also injected into the intact spinal cord at the level of Th10, in one mouse each. These mice were used for autoradiography and histological analyses.

SCI Animal Model and hiPSC-NS/PCs Transplantation

Adult female NOD-SCID mice (8-weeks-old, 20–22 g; Clea, Tokyo, Japan) were anesthetized intraperitoneally (i.p.) with ketamine (100 mg/kg) and xylazine (10 mg/kg). After laminectomy at the level of 10th thoracic spinal vertebra, the dorsal surface of the dura mater was exposed, and contusive SCI was induced using an IH impactor (a force defined impact (60 kdyn) with a stainless steel-tipped impactor; Precision Systems and Instrumentation, Lexington, KY, USA). Donor cells were prepared *in vitro* from 253G1-NS/PCs. Nine days after SCI, 253G1-NS/PCs were transplanted into the lesion epicenter of each mouse with a glass micropipette at a rate of 1 µl/min using a Hamilton syringe (25 µl) and a stereotaxic micro injector (KDS 310; Muromachi Kikai Co. Ltd., Tokyo, Japan).

Bioluminescence Imaging

A Xenogen-IVIS spectrum cooled charge-coupled device optical macroscopic imaging system (PerkinElmer, Waltham, MA) was used for bioluminescence imaging (BLI) to monitor the survival of the transplanted hiPSC-NS/PCs and U-251MG. D-luciferin (VivoGlo Luciferin; Promega, Madison, WI) was injected into the right subcutaneous limb at a dose of 0.3/kg body weight. Animals were placed in a light-tight chamber, and photons emitted from luciferase-expressing cells were collected with

integration time of five seconds to one minute for each image. BLI signals were quantified as maximum radiance units (photons/sec/cm^2/steradian) and presented as $\log_{10}$ (photons / sec) values. BLI imaging was performed weekly up to 14 days on U-251MG-implanted mice (n = 5) because of their survival periods, and 253G1-NS/PCs- and 414C2-NS/PCs-implanted mice (n = 5, each), up to 28 days after transplantation.

PET and CT Scanning

Prior to PET scanning, mice were anesthetized with 5% (v/v) isoflurane and a custom made catheter with needle was inserted into the tail vein for the injection. Subsequently, a mouse maintaining anesthesia with 1.5% (v/v) isoflurane was secured on a custom-designed chamber and placed in a PET scanner (Inveon). The body temperature of the mouse was maintained with a heated water (38–40 °C) circulation system (T/Pump TP401; Gaymar Industries, Orchard Park, NY, USA). Dynamic emission scans were performed in a three-dimensional list mode for 30 min (1 min × 4 frames, 2 min × 8 frames, and 5 min × 2 frames) after a bolus injection of [^{18}F] FEDAC (13–15 MBq/head). To determine the anatomical localization of the brain, the chamber with the mouse was moved to a small-animal CT system (CosmoScan GX) under anesthesia with 2–3% (v/v) isoflurane after the PET scan. CT images were acquired on non-enhanced scans for 16 seconds using the X-ray source set at 70 kVp and 114 µA. To acquire tissue time-activity curves (tTACs) of [^{18}F] FEDAC, dynamic PET images and average CT attenuation images were fused using PMOD software (version 3.4, PMOD Technologies, Zurich, Switzerland) and, subsequently, volumes of interest (VOIs) were manually drawn on the ipsilateral and contralateral sides. The uptake of radioactivity in the brain was decay-corrected to the injection time and expressed as SUV, which was normalized to the injected radioactivity and body weight. The SUV was calculated as follows: SUV = (radioactivity per milliliter tissue/injected radioactivity) × gram body weight. The area under the activity curve (AUC, SUV × minutes) of [^{18}F] FEDAC in the brain was calculated from 10 to 30 min after the injection on tTACs.

Production of [^{18}F] FEDAC

[^{18}F] FEDAC (n = 7) was prepared via direct [^{18}F] fluorination by reaction (Kryptofix 222, 110 °C, 15 min) with the tosylated precursor and [^{18}F] fluoride. The radiochemical purity and molar activity of [^{18}F] FEDAC were over 99% and 294 ± 64 GBq/µmol at the

end of synthesis, respectively.

<u>MRI</u>

All MRI experiments were performed using a 7.0 - T MRI scanner (magnet, Kobelco and JASTEC, Kobe and Tokyo Japan; console, Bruker Biospin, Ettlingen, Germany) with a volume coil for transmission (Bruker Biospin) and a quadrature coil for reception (Rapid Biomedical, Rimpar, Germany). 253G1- and 414C2- NS/PCs grafted mice (n = 1, each) were anesthetized with 2.0% isoflurane gas (Escain, Mylan, PA, USA) and a 1:5 = O_2: room-air mixture during MRI scanning. Rectal temperature was measured with an optical fiber thermometer (FOT-L, FISO Technology, Quebec City, QC, Canada) inserted into the rectum, and maintained at 36.5 ± 0.5 °C by a warm water pad and warm air heating system. Body temperature and breathing were monitored using a Biopac system (BIOPAC Systems, Goleta, CA, USA). Multi-slice T1-weighted images (T1W) were acquired using a multi-slice multi-echo (MSME) sequence. The imaging parameters used were repetition time (TR)/echo time (TE) = 400/8.6 ms, field of view (FOV) = 19.2 × 15.0 mm, matrix = 192 × 150, slice thickness = 0.60 mm, slice gap = 0.1 mm, number of excitations (NEX) = 12, and scan time = 12 min 0 seconds. Multi-slice T2 were acquired using a rapid acquisition with relaxation enhancement (RARE) sequence. The imaging parameters used were TR/TE = 3000/30 ms, RARE factor = 4, FOV = 19.2 × 15.0 mm, matrix = 192 × 150, acquisition matrix size = 256 × 192 (Zero-Fill Acceleration = 1.34), slice thickness = 0.60 mm, slice gap = 0.1 mm, NEX = 4, and scan time = 5 min 36 sec. In all images, a fat-saturated pulse was used to exclude chemical-shift artifacts. All MR images were analyzed using Osirix (Pixmeo, Bernex, Switzerland) image analysis software.

<u>*Ex vivo* autoradiography</u>

All mice were deeply anesthetized and injected intravenously with [^{18}F] FEDAC (12–14 MBq/head), and transcardially perfused with 4% paraformaldehyde (PFA; pH 7.4) after 30 min. The brains and spinal cords were quickly removed and frozen with dry-ice powder. Frozen brain and spinal cord sections (20-µm-thick) were prepared using a microtome (HM560; Carl Zeiss, Oberkochen, Germany) and thaw mounted on glass-slides (Matsunami Glass, Tokyo, Japan). The sections were exposed to the imaging plate (BAS-MS2025; FUJIFILM, Tokyo) for 1 h. Radioactivity in the brain or spinal cord was detected by scanning the imaging plate using a BAS-50000 system (FUJIFILM).

Histological Analyses

The brain and spinal cord sections after autoradiography were used for immunohistochemical analyses. The sections were stained with the following primary antibodies: anti-GFP (600-101-215, goat IgG, 1:200; Rockland Immunochemicals, Gilbertsville, PA, USA), anti-human cytoplasm (STEM121, Y40410, mouse IgG1, 1:200; Takara Bio, Kusatsu, Japan), anti-human-specific TSPO (NP157, rabbit IgG, 1:300; National Institute for Quantum and Radiological Science and Technology, Chiba, Japan), anti TSPO (ab118913, goat IgG, 1:200; Abcam, Cambridge, UK), anti-Hu (human IgG; 1:1000; the Rockefeller University, New York, NY, USA), anti-Iba1 (GTX100042, rabbit IgG, 1:500; GeneTex Inc., Irvine, CA, USA), anti-glial fibrillary acidic protein (GFAP, 13-0300, rat IgG2a, 1:200;ThermoFisher Scientific, Rockford, USA), anti-human-specific Nestin protein (MAB5326, mouse IgG1, 1:500; Merck Millipore, Billerica, Massachusetts, USA). Nuclei were stained with Hoechst 33258 (10 µg/mL; Sigma). The samples were examined on an inverted fluorescence microscope (BZ-9000, Keyence, Osaka, Japan) or a confocal laser-scanning microscope (LSM700, Carl Zeiss, Munch, Germany).

Microarray analysis

The microarray data was previously reported[1]. Briefly, the injured mice were anesthetized and transcardially perfused with heparinized saline (5 U/ml) at 9 dpi or 42 dpi (n = 3, each). Dissected segments of spinal cord at the Th10 level were rapidly frozen and placed in TRIzol (Invitrogen). Total RNA was isolated using an RNeasy Mini Kit (Qiagen Inc., Hilgen, Germany), in accordance with the manufacturer's instructions. As a control, samples of naïve spinal cord were harvested by the same protocol. For microarray analysis, RNA quality was assessed using a 2100 Bioanalyzer (Agilent Technologies Inc., Santa Clara, CA, USA), and 100 ng of total RNA was reverse transcribed, biotin labeled, and hybridized to a GeneChipW Mouse Genome 430 2.0 Array (Affymetrix Inc., Santa Clara, CA, USA). The array was then washed and stained in a Fluidics Station 450, according to the manufacturer's instructions[2]. The microarrays were scanned using a GeneChip Scanner 3000 7G, and the raw image files were converted to normalized signal intensity values using the MAS 5.0 algorithm.

SI Figures and Figure legends

Fig. S1.

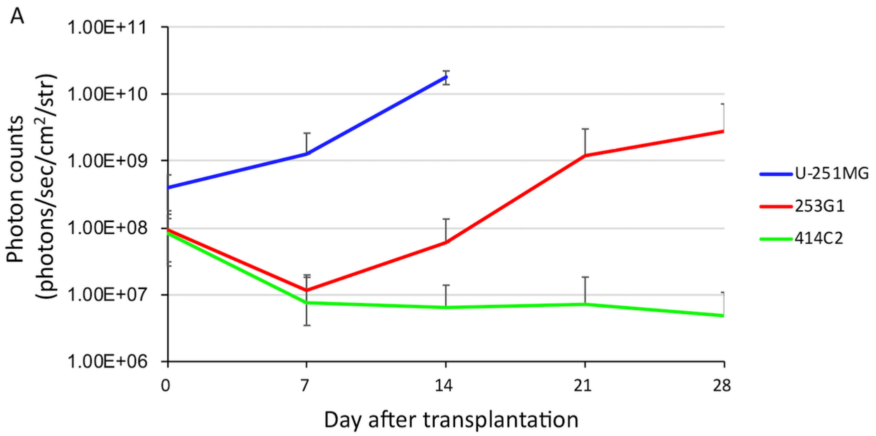

Bioluminescence tracking of the transplanted hiPSC-NS/PCs. (A): Quantitative analyses of the photon counts derived from the grafted cells for four weeks (the control U-251MG group was evaluated for two weeks due to their survival period). The grafted 253G-NS/PCs proliferated more rapidly than that of the 414C2-NS/PCs. Abbreviations: hiPSC-NS/PCs, human induced pluripotent stem cells derived neural stem/progenitor cells.

Fig. S2.

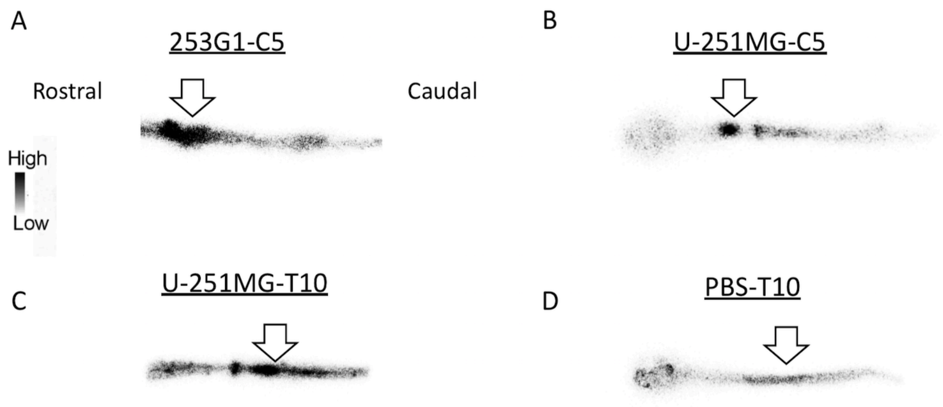

***Ex vivo* autoradiography with [^{18}F] FEDAC in the 253G1-NS/PCs-grafted mouse spinal cords. (A-D)**: Representative autoradiography images of the 253G1-NS/PCs, U-251MG or PBS grafted mouse spinal cords after injection of the [^{18}F] FEDAC. C5 level of the spinal cord grafted with 253G1-NS/PCs **(A)**; and U-251MG **(B)**; T10 level of the spinal cord grafted with U-251MG **(C)**; and PBS **(D)**. Arrows indicate the transplanted site. Abbreviations: NS/PCs, neural stem/progenitor cells; PBS, phosphate-buffered saline.

Fig. S3.

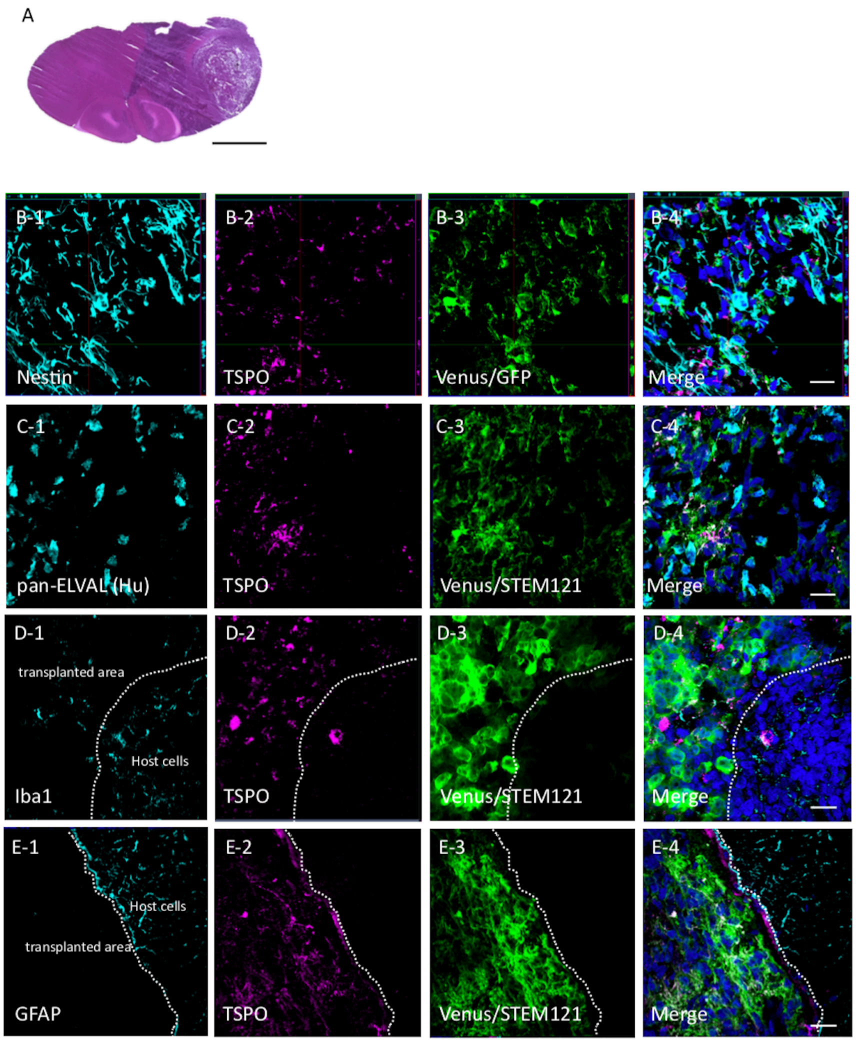

Histological analyses of the 253G1-NS/PCs-grafted mouse brains (related to Fig. 6). (A): Representative hematoxylin and eosin image of the coronal section 56 days post-transplantation. The grafted cells were labeled with Venus/GFP/anti-human cytoplasm $(STEM121)^+$ and TSPO/Nestin **(B)**; TSPO/pan-ELAVL (Hu) (a human specific neuron marker) **(C)**; TSPO/Iba1 (microglia) **(D)**; TSPO/GFAP (astrocyte) **(E)**. The nuclei were stained with Hoechst 33258. Scale bars, 1000μm in (A), 20μm in **(B-E)**. Abbreviations: NS/PCs, neural stem/progenitor cells; GFP, green fluorescent protein; GFAP, glial fibrillary acidic protein.

Fig. S4.

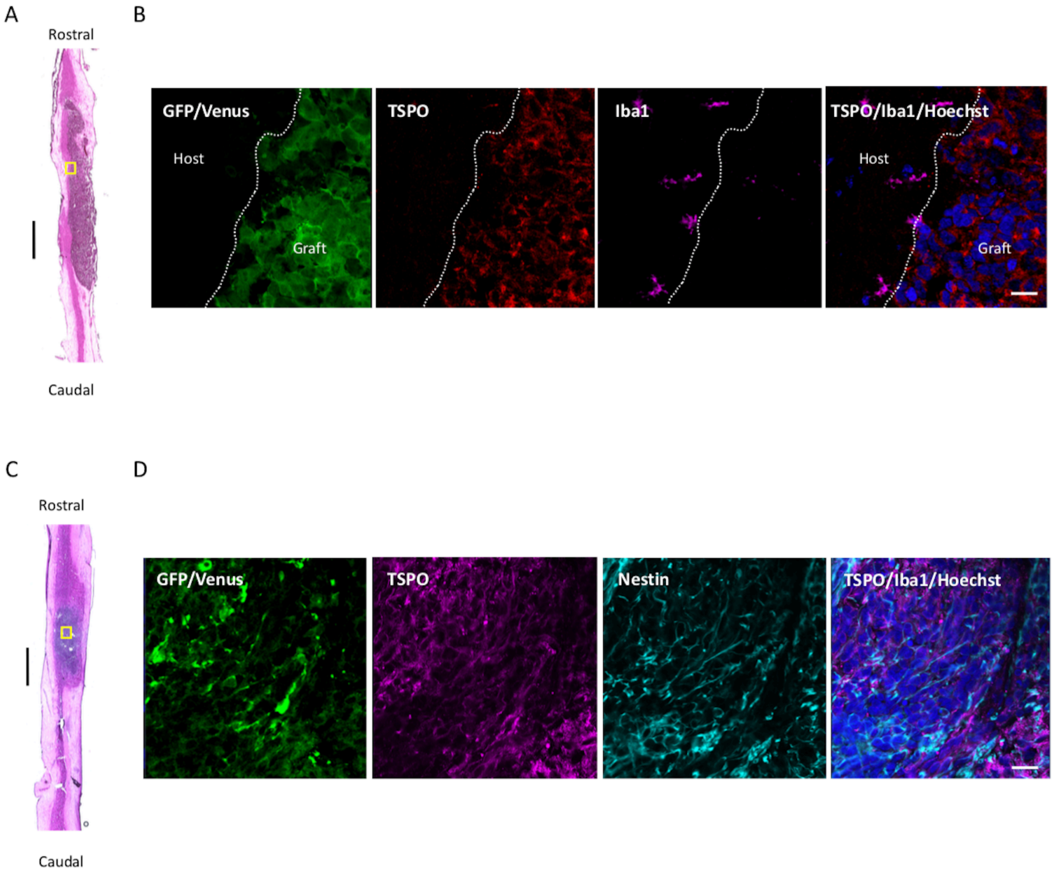

Histological analyses of the U-251MG- or 253G1-NS/PCs-grafted intact spinal cords of NOD/SCID mice. (A): Representative hematoxylin and eosin (H &E) sagittal image of the U-251MG-grafted spinal cord section 21 days post transplantation. **(B):** Representative image of the U-251MG- grafted spinal cord section immunostained with Venus/GFP, TSPO and Nestin. **(C):** Representative H &E sagittal image of the 253G1-NS/PCs-grafted spinal cord sections 56 days post-transplantation. **(D):** Representative image of the 253G1-NS/PCs-grafted spinal cord section immunostained with Venus/GFP, TSPO and Nestin. The nuclei were stained with Hoechst 33258. Scale bars, 1000μm in **(A and C)**, 20μm in **(B and D)**. Abbreviations: NS/PCs, neural stem/progenitor cells; GFP, green fluorescent protein.

Fig. S5.

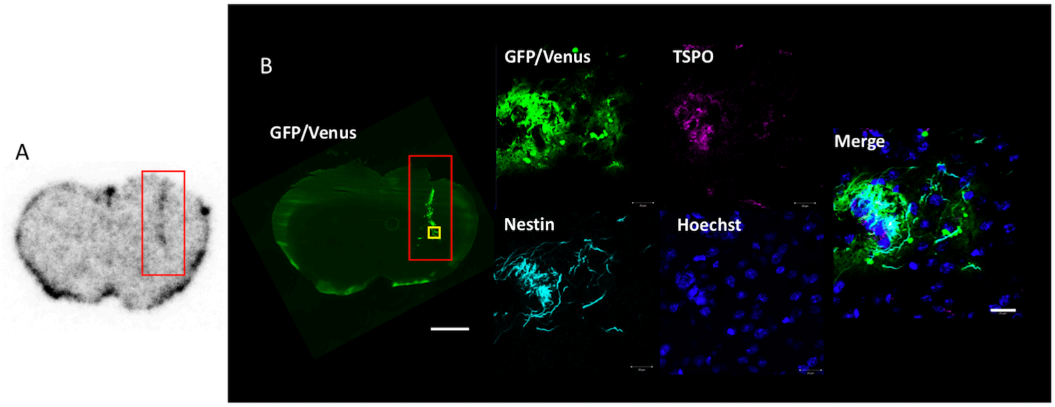

***Ex vivo* autoradiography with [^{18}F] FEDAC and immunohistological analyses on the 414C2-NS/PCs-grafted brain of NOD/SCID mice. (A)**: Representative coronal images of 414C2-NS/PCs-grafted brain sections in *ex vivo* autoradiography with [^{18}F] FEDAC (red box indicates the graft area). **(B)**: Magnified regions are indicated by the yellow box, showing representative coronal images of the 414C2-NS/PCs-grafted mouse brain sections immunostained with Venus/GFP, TSPO and Nestin. The nuclei were stained with Hoechst 33258. Scale bars, 1000μm in (A), 20μm in **(B)**. Abbreviations: NS/PCs, neural stem/progenitor cells; GFP, green fluorescent protein.

Fig. S6.

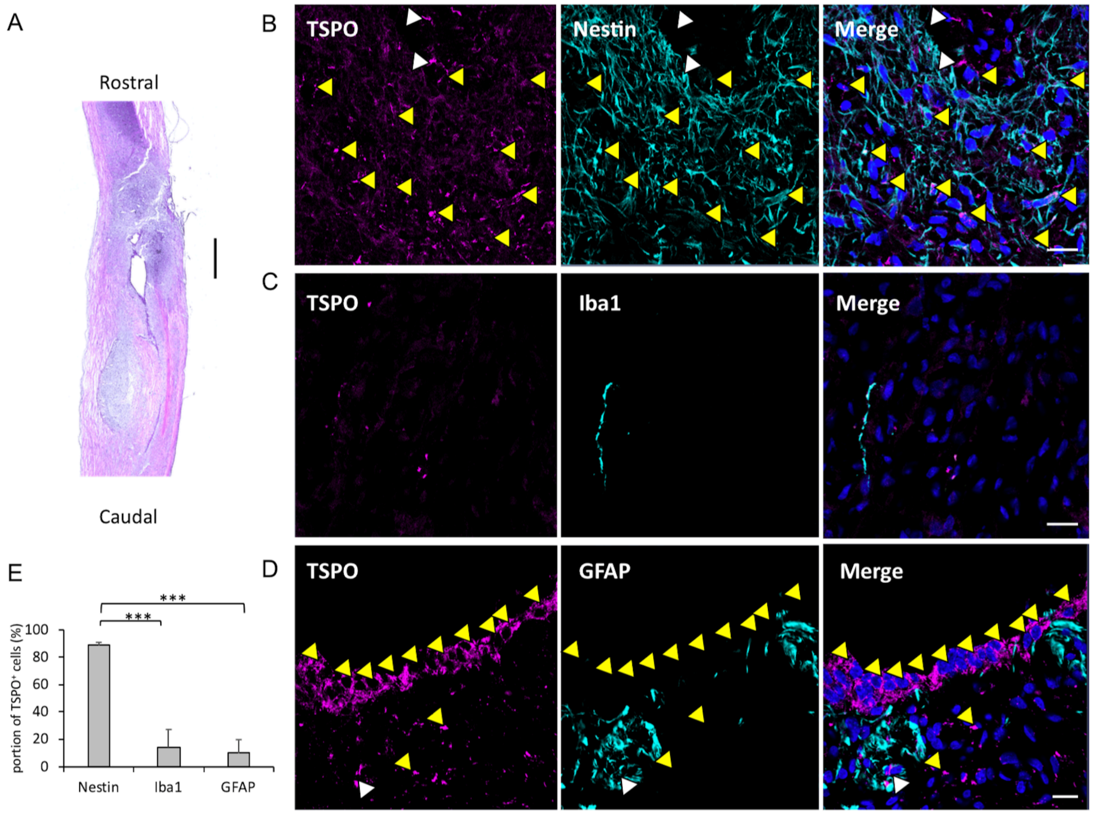

TSPO expression in immature neural cells in 253G1-NS/PCs-grafted injured spinal cord of NOD/SCID mice (103 days post transplantation). In the present study, TSPO immunostaining was newly performed for specimen derived from 253G1-NS/PCs-grafted NOD/SCID mice SCI models, which were generated in our previous studies[3]. **(A)**: Representative hematoxylin and eosin sagittal image of 253G1-NS/PCs-grafted inured spinal cord. **(B-D)**: Representative images of immunohistochemical staining for each cell-specific type markers. TSPO/Nestin (yellow arrowheads indicate $TSPO^+/Nestin^+$ cells while white arrow heads indicate $TSPO^+/Nestin^-$ cells; TSPO/Iba1 (microglia) **(C)**; TSPO/GFAP (astrocyte) (yellow arrowheads indicate $TSPO^+/GFAP^-$ cells while white arrow heads indicate $TSPO^+/GFAP^+$ cells **(D)**. **(E)**: Bar graph showing the percentage of $TSPO^+$ cells for each cell-specific marker; Nestin, Iba1 and GFAP. The nuclei were stained with Hoechst 33258. Scale bars, 1000μm in (A), 20μm in **(B-D)**. Values are means ± SD (n = 3). $^{***}P < 0.001$ according to one-way ANOVA with the Tukey-Kramer test. Abbreviations: NS/PCs, neural stem/progenitor cells; GFAP, glial fibrillary acidic protein.

Fig. S7.

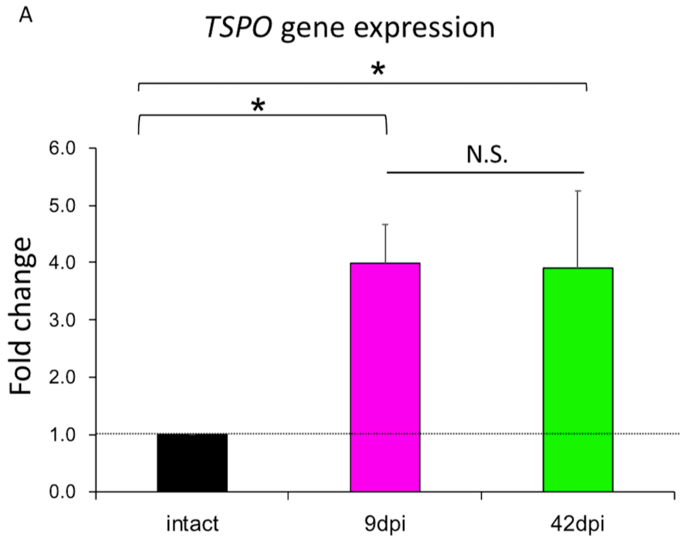

Temporal changes of *TSPO* mRNA expression after the SCI（9 dpi and 42 dpi）by the re-analysis of microarray data[1]. Microarray analysis revealed that *TSPO* mRNA expression reached its peak within six weeks post-SCI in mouse models. (A): The microarray data revealed the gene expression signals of *TSPO* at 9 dpi and 42 dpi groups compared with the intact group (equal to 1). *TSPO* mRNA was significantly up-regulated at 9 dpi and there was no significant difference between 9 dpi and 42 dpi. The data shows the mean fold-change values versus intact samples. Values are means ± SD (n = 3). *$p < 0.05$ and not significant (N.S.) according to one-way ANOVA with the Tukey-Kramer test. Abbreviations: SCI, spinal cord injury; dpi, day after spinal cord injury.

Table.S1. (related to Fig.3). DVR of the grafted area in each group (253G1 group, n=5; 414C2 group n=4; U251MG group, n=5 and PBS group, n=4). Abbreviations: DVR, distribution volume ratio

Group	DVR (mean ± SD)		
253G1	1.3	±	0.1
414C2	1.0	±	0.1
U-251MG	3.1	±	0.7
PBS	1.0	±	0.1

Supplemental References

1. Nishimura S, Yasuda A, Iwai H, et al. Time-dependent changes in the microenvironment of injured spinal cord affects the therapeutic potential of neural stem cell transplantation for spinal cord injury. Mol Brain. 2013;6:3.
2. Lockhart DJ, Dong H, Byrne MC, et al. Expression monitoring by hybridization to high-density oligonucleotide arrays. Nat Biotechnol. 1996;14:1675-1680.
3. Nori S, Okada Y, Nishimura S, et al. Long-term safety issues of iPSC-based cell therapy in a spinal cord injury model: oncogenic transformation with epithelial-mesenchymal transition. Stem Cell Reports. 2015;4:360-373.
